# Using network analysis to explore the validity and influential items of the Parkinson’s Disease Questionnaire-39

**DOI:** 10.1038/s41598-023-34412-4

**Published:** 2023-05-03

**Authors:** Aline Schönenberg, Diego Santos García, Pablo Mir, Jian-Jun Wu, Konstantin G. Heimrich, Hannah M. Mühlhammer, Tino Prell

**Affiliations:** 1 Department of Geriatrics, Halle University Hospital, Halle (Saale), Germany; 2 Department of Neurology, Complejo Hospitalario Universitario de A Coruña (CHUAC), A Coruña, Spain; 3 Unidad de Trastornos del Movimiento, Servicio de Neurología y Neurofisiología Clínica, Instituto de Biomedicina de Sevilla, Hospital Universitario Virgen del Rocío/Consejo Superior de Investigaciones Científicas/Universidad de Sevilla, Seville, Spain; 4 Centro de Investigación Biomédica en Red Sobre Enfermedades Neurodegenerativas, Madrid, Spain; 5 Departamento de Medicina, Facultad de Medicina, Universidad de Sevilla, Seville, Spain; 6 Department of Neurology and National Clinical Research Center for Aging and Medicine, Huashan Hospital, Fudan University, Shanghai, China; 7 Department of Neurology, Jena University Hospital, Jena, Germany

**Keywords:** Neurological disorders, Medical research

## Abstract

Quality of life (QoL) in people with Parkinson´s disease (PD) is commonly measured with the PD questionnaire-39 (PDQ-39), but its factor structure and construct validity have been questioned. To develop effective interventions to improve QoL, it is crucial to understand the connection between different PDQ-39 items and to assess the validity of PDQ-39 subscales. With a new approach based on network analysis using the extended Bayesian Information Criterion Graphical Least Absolute Shrinkage and Selection Operator (EBICglasso) followed by factor analysis, we mostly replicated the original PDQ-39 subscales in two samples of PD patients (total N = 977). However, model fit was better when the “ignored” item was categorized into the *social support* instead of the *communication* subscale. In both study cohorts, “depressive mood”, “feeling isolated”, “feeling embarrassed”, and “having trouble getting around in public/needing company when going out” were identified as highly connected variables. This network approach can help to illustrate the relationship between different symptoms and direct interventional approaches in a more effective manner.

## Introduction

Parkinson’s disease (PD) is a chronic progressive neurodegenerative disease characterized by motor- and non-motor symptoms affecting quality of life (QoL). Several scales have been proposed to assess QoL of people with PD^1^. Among the existing measurements, the 39-item Parkinson's Disease Questionnaire (PDQ-39) is the most thoroughly tested and applied questionnaire, and it is recommended to assess health-related QoL in PD^2^. The PDQ-39 was developed in 1995 by Peto et al.^3,4^ and consists of 39 items, divided into 8 subscales: mobility (MOB, 10 items), activities of daily living (ADL, 6 items), emotional well-being (EMO, 6 items), stigma (STI, 4 items), social support (SOC, 3 items), cognition (COG, 4 items), communication (COM, 3 items), and bodily discomfort (BOD, 3 items). For each item, answers are given on a 5-point Likert scale: never, occasionally, sometimes, often, and always. The scores from each domain are computed into a score ranging from 0 (best) to 100 (worst). In addition, a single index was described as overall measure of health-related QoL^4^. The frequently translated PDQ-39 is overall considered reliable, valid, comprehensive, and sensitive to change^1,2^. However, several important psychometric issues regarding details of the PDQ‐39 have not been sufficiently addressed. In particular, studies addressing the validity of the PDQ-39’s dimensionality have shown inconclusive results^5–7^,with items’ group assignment failing to match the assumed PDQ‐39 subscales, and eigenvalues of several factors only marginally exceeding 1^7^. The PDQ-39's scale reliability was also suboptimal for SOC, COG and BOD^7^. Considering these aspects is important, as reliability is crucial in clinical studies using the PDQ-39 as a measure. In addition to an overall sum score, it is critical to comprehend what the subscores represent. This is a matter of construct validity. For instance, in previous studies, the COG subscale was more closely linked to depression than to neurocognitive tests^8^ or with sleep disturbances and hallucinations^9^. Therefore, additional studies are needed to assess the construct validity of the PDQ-39 to understand the item allocation to the subscales, as well as their relation to each other.

Network analysis is a relatively new and promising method for modeling interactions between large numbers of variables. Instead of trying to reduce the structure of the variables to their shared information (such as a latent variable), as is done in abovementioned modeling, the network estimates the relation between all variables directly^10–12^. Network elements (e.g., items of a score or symptoms) are conceptualized as elements of a complex interacting dynamical system^11^. Networks consist of *nodes* representing observed variables, and the relationships (formally called *edges*) between these nodes. Instead of visualizing the relationships of all variables with each other, networks can be estimated using regularization techniques originating from the field of machine learning. Regularization means that edges that are likely to be spurious are removed from the model, leading to networks that are sparse and easier to interpret^10^. Beyond the more conventional methods, this network approach has the added benefit of mapping out the connections between different variables in the network and thus helps understand which symptoms are linked. It may also provide an understanding of how sub-scales of a questionnaire are connected and which items are cross-domain bridges, thus providing richer information about the questionnaire structure^10,13^.

The purpose of the current investigation was two-fold. First, we applied network analysis followed by confirmatory factor analyses (CFA) to evaluate the connection between PDQ-39 items and their subscales. Specifically, we attempted to comprehend whether the subsections of the PDQ-39 are embodied in the item groupings in the network and which items act as links between the various subsections. We especially employed network analysis to attain insight in the cross-domain linkages and to detect evidence for substitute item groupings. Secondly, we sought to gain further understanding into the connectedness of distinctive PDQ-39 items and their purported role as targets for interventions to raise quality of life in PD.

## Results

### COPPADIS study

**Figure 1:**
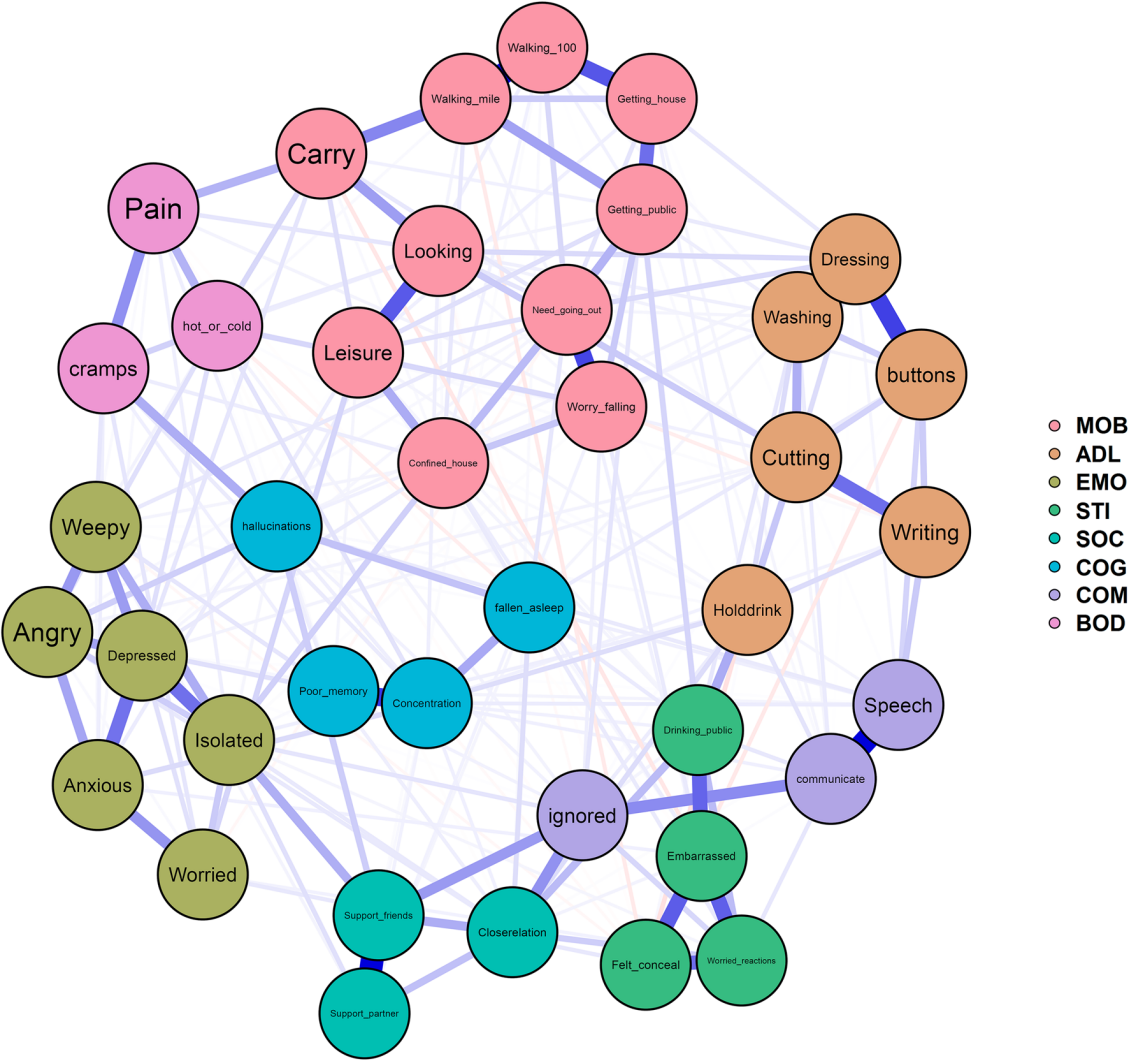
Network plot. Abbreviations: mobility (MOB), activities of daily living (ADL), emotional well-being (EMO), stigma (STI), social support (SOC), cognition (COG), communication (COM), and bodily discomfort (BOD). Leisure (Leisure activities), Looking (Looking after home), Carry (Carry shopping bags), Walking_mile (Walking half a mile), Walking_100 (Walking 100 yards), Getting_house (Getting around the house), Getting_public (Getting around in public), Need_going_out (Need company when going out), Worry_falling (Worry falling in public), Confined_house (Confined to the house), Washing (Washing), Dressing (Dressing), buttons (Do buttons or shoe laces), Writing (Writing clearly), Cutting (Cutting food), Holddrink (Hold a drink without spilling), Depressed (Depressed), Isolated (Isolated and lonely), Weepy (Weepy or tearful), Angry (Angry or bitter), Anxious (Anxious), Worried (Worried about the future), Felt_conceal (Felt need to conceal PD), Drinking_public (Avoid eating/drinking in public), Embarrassed (Embarrassed due to PD), Worried_reactions (Worried people's reactions), Closerelation (Close relationships), Support_partner (Support from partner), Support_friends (Support from family or friends), fallen_asleep (Unexpectedly fallen asleep), Concentration (Concentration), Poor_memory (Poor memory), hallucinations (Dreams or hallucinations), Speech (Speech), communicate (Unable communicate properly), ignored (Felt ignored), cramps (Painful cramps or spasms), Pain (Pain in joints or body), hot_or_cold (Unpleasantly hot or cold).

**Figure 2:**
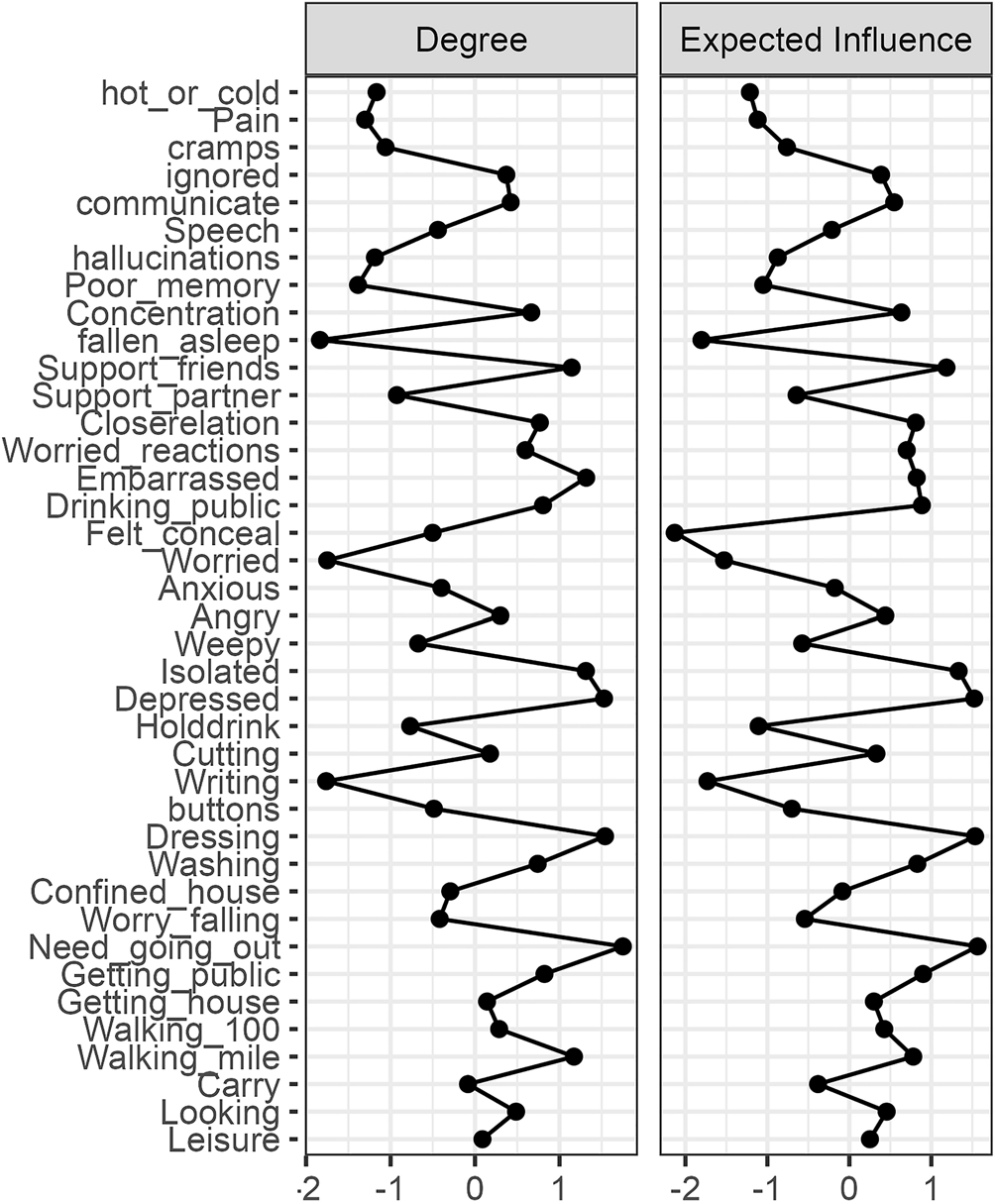
Centrality plot. Abbreviations: Leisure (Leisure activities), Looking (Looking after home), Carry (Carry shopping bags), Walking_mile (Walking half a mile), Walking_100 (Walking 100 yards), Getting_house (Getting around the house), Getting_public (Getting around in public), Need_going_out (Need company when going out), Worry_falling (Worry falling in public), Confined_house (Confined to the house), Washing (Washing), Dressing (Dressing), buttons (Do buttons or shoe laces), Writing (Writing clearly), Cutting (Cutting food), Holddrink (Hold a drink without spilling), Depressed (Depressed), Isolated (Isolated and lonely), Weepy (Weepy or tearful), Angry (Angry or bitter), Anxious (Anxious), Worried (Worried about the future), Felt_conceal (Felt need to conceal PD), Drinking_public (Avoid eating/drinking in public), Embarrassed (Embarrassed due to PD), Worried_reactions (Worried people's reactions), Closerelation (Close relationships), Support_partner (Support from partner), Support_friends (Support from family or friends), fallen_asleep (Unexpectedly fallen asleep), Concentration (Concentration), Poor_memory (Poor memory), hallucinations (Dreams or hallucinations), Speech (Speech), communicate (Unable communicate properly), ignored (Felt ignored), cramps (Painful cramps or spasms), Pain (Pain in joints or body), hot_or_cold (Unpleasantly hot or cold).

The network plot based on data from the COPPADIS study (N = 694 PD patients, see Supplement Table 1) is displayed in Fig. 1 and its corresponding centrality measures are shown in Fig. 2 (tabulated in detail in Supplement Table 2). Visually, the network can be divided into several domains that mostly fit to the proposed PDQ-39 subscales by Peto et al.^3^. The thickest edges were found within the respective domains, although the borders between these domains were bridged by various cross-domain associations. For example, the item “isolated” connects the items in the EMO subscale to the COC and SOC subscales.

When examining strength as a centrality measure, high node strength indices were seen for "need going out", "dressing", "depressed", and "isolated". After performing the bootstrapped centrality difference test (Supplement Table 3), it became evident that the most influential items were not different from one another in terms of their centrality, so no definitive order can be determined. Nevertheless, the items "need_going_out", "dressing", "depressed", "isolated", "walking_mile", "embarrassed", "Support_friends", "close_relation", "getting_public", and "drinking_public" were identified as being the most connected within the network. Alterations in the value of these points thus have a bearing on other nodes within the network. Conversely, items such as "anxious", "illusions" and "hot_or_cold" (unpleasantly hot or cold) appear to be less connected when taking into consideration their strength as opposed to other nodes. The expected influence also reveals that the items "require going out", "getting dressed" and "depressed" are profoundly influential and well-connected nodes within the network^14^.

In terms of validity, it is worth to note that according to the network, some items were connected to different subscales. For example, “ignored” showed connections to both “communicate” (COM) and “support friends”/“close relations” (SOC), as can be seen in the network plot and the edge weights in Supplement Table 3. This can also be seen by a low item-to-rest correlation of r = 0.386 of “ignored” to its subscale COM (Supplement Table 4). Also “hold drink” from the ADL subscale is more strongly connected to “drinking public” from the STI subscale than to the items of the ADL scale. “Hallucinations” are closer related to the “cramp” item from BOD than to the items of its COG subscale. For an overview of detailed edge strengths see Supplement Table 2. Accordingly, a CFA revealed better model fit when “ignored” was categorized into the SOC subscale than into the COM subscale (Supplement Tables 5 and 6).

Supplement Figs. 1–3 show the results of the accuracy and stability checks. Overall, the network model is stable, many of the identified edges and centrality measures are significantly different from each other. The case-dropping bootstrap procedure shows that CS-Cs of node strength and edges were 0.67 and 0.75, respectively.

### Chen et al. study

A second sample from Chen et al. (N = 283, see Supplement Table 1 for a comparison of both study groups) was used to confirm the findings of the COPPADIS study^15^. Here, the network plot (Supplement Fig. 4) was overall comparable to the COPPADIS study, although visual differences exist between the two network plots. In the second network, strength was again high for “depressed” as well as “embarrassed”, “communication”, “isolated”, “getting_public”, “need_going_out”, “angry”, “anxiety”, “embarrassed”, and “dressing” (Supplement Fig. 5). When looking at centrality different tests using bootstrapping methods, node strength did not differ significantly between the items with highest strength listed above, suggesting that they all have a large amount of relevant connections. Again, the item “ignored” showed connections to both “communicate” (COM) and “support friends” / “close relations” (SOC). This was also confirmed by CFA where the model fit was again better when “ignored” was categorized into SOC subscale than into the COM subscale (Supplement Table 7 and 8). Although the sample size of the study by Chen et al. was lower than the COPPADIS study, the network can still be regarded as stable and interpretable (Supplement Figs. 6–8). The case-dropping bootstrap procedure shows that CS-Cs of node strength and edge were 0.52 and 0.52, respectively.

## Discussion

As far as we are aware, this is the first exploration to utilize network analysis on the PDQ-39. Network analysis can shed light on the theoretical structure of QoL and can expose potential targets for enhancement in clinical intervention studies^16^. Our study has two significant results in terms of our research goals. Firstly, we used multiple methodological techniques to display that the item "ignored" has a closer relation to the SOC subscale rather than to COM. Secondly, we identified network hubs that bring to light symptoms that have a strong connection within the network and can likely have an impact on the QoL in PD All in all, although both CFA and network analysis render similar results in terms of the composition of subscales, they both bring forth beneficial knowledge to the examination of data. Network analysis evaluates the interconnection between items instead of determining their particular impact on a shared latent variable. This incorporates mapping the movement of data via, for instance, a third item and furnishes extra visual and statistical details on how items and subscales of a survey are intertwined. Consequently, CFA and network analysis can either be in agreement or not. Therefore, network analysis may help direct interventions by mapping the flow of association between the included items^12,16^. In both study cohorts, depressive mood, feeling isolated, feeling embarrassed, and having trouble getting around in public/needing company when going out were identified as well-connected variables. This approach can help identify important aspects of the illness and direct interventional approaches in a more effective manner.

The PDQ-39 assesses different symptoms and functions with a Likert-scaled rating. It is worth to note that the symptom centrality and the mean value of the symptom are different aspects and constructs which are only weakly associated^17^. For example, the value of a PDQ-39 item can change without relevant changes within the network, because the network portrays the connections *between* items and the influence they have on each other in terms of co-occurrence. Thus, different conclusions about single PDQ-items or subscales can be drawn when looking at centrality measures or symptom severity of PDQ-39 items^18^. For example, “depressive” and “need company when going out” both have high strength in the network. However, mean value for “need company when going out” was lower than for “depressive” (Supplement Table 1). Items in the PDQ-39 that were determined as being central in the network are well-connected items that are linked to many other symptoms of the disease, thus they may be considered relevant to the whole concept of QoL, but see also the limitations section for the interpretation of network analysis. Consequently, aiming at these items specifically could potentially result in more successful and efficient interventions, even though it is only possible to confirm the causal inference with clinical trials and long-term data^16,19^. Given the restricted resources in medical care and the need for efficient therapies, findings from network analysis can support the development of effective and tailored interventions, as it visually and statistically displays which items are linked; this provides researchers with a helpful starting point to develop tailored hypotheses^19^. Our findings from the COPPADIS study data points to four areas that should be the focus of attention; these include “needing company when going out”, “dressing”, “isolated” and treating depression as these were strongly interlinked in the network. Depression in particular has previously been identified as a predictor of poorer QoL in PD^20^. However, it is interesting that focusing on “dressing” (high centrality) rather than on “cutting” or “writing” (low centrality) as other aspects of daily activities appears to have a faster effect on well-being and overall QoL in this group of PD patients. Of course, findings from one cohort cannot be fully generalized to other cohorts of PD patients, as attitudes, beliefs, and aspects regarded as important might differ from country to country. This can be seen by slight network differences between the Spanish COPPADIS cohort and the Chinese cohort. While “depressed” can be considered highly connected in both cohorts, “communication” for example was more well-connected in the Chinese than in the Spanish cohort. Although cultural aspects may play a role for these differences, it is worth to mention that also PD-related characteristics markedly differed between both cohorts. People in the Chinese cohort were younger, had poorer motor function, more depressive symptoms and poorer cognitive function (Supplement Table 1).

Concerning the influence of depression on QoL, for future studies, it would be promising to additionally include measures of depression in the network analysis in order to understand which depressive symptoms are closely linked with QoL in PD patients. Also the inclusion of other PD-related factors, such as motor function or disease stage, to a network could provide useful insight to gain better understanding of QoL and treatment options in PD. For instance, if we know how anxiety or other non-motor symptoms are associated with QoL in networks, we could develop targeted and more efficient interventions with better outcomes. However, it is worth to note that at the current state of its methodical development, network analysis is just one technique beside other explorative approaches and needs justification by confirmatory analysis methods.

Additionally, using several approaches we found that “ignored” seems to be more closely related to the SOC subscale rather than the COM subscale. This was demonstrated in the network analyses and confirmed by CFA as well as Cronbach’s Alpha in both samples. This is worth to note as it influences the validity of the separate PDQ-39 subscales and supports that evidence regarding the psychometric properties of some of the PDQ-39 subscales is questionable^8^. The PDQ-39 was initially developed from a 65-item questionnaire, which was tested in a larger sample. A factor analysis from this sample found 10 factors, and 2 factors were removed by the authors because they were regarded as repetitive^3^. However, several studies have shown that some items do not correlate well with their respective subscales^5,7,8^. In particular, items from the COG subscale frequently correlate more strongly with other domains^7,8^. This was also the case in our analysis which suggests that “hallucinations” are associated with BOD items rather than with the other COG items. This means that the PDQ-39 subscales should be used with caution as outcome measure for health-related QoL in PD trials.

### Limitations

Our study has several limitations. As explained, centrality measures of respective PDQ-39 items should be generalized with caution, as they might depend on the studied cohort (e.g., different inclusion and exclusion criteria etc.) and their clinical and cultural background. Additionally, the interpretation of centrality indices for psychological data is highly debated, which is why we opted against reporting all available indices (such as betweenness or closeness, as these are not recommended for psychological data)^19,21,22^. However, when assessing strength and expected influence, we adhered to the steps described by Epskamp et al.^10^ to ensure interpretability of the results. This includes using bootstrapping methods to assess the stability and significant difference of indices. However, while centrality indices can be useful to identify important areas of an illness or a psychosocial construct to improve effectiveness of interventions, they should be interpreted with caution and should be taken as an explorative approach. As Dablander and Hinne^19^ state, in network analysis—especially in undirected networks—it is difficult to assess the *direction* of information flow through the network. Unless a network is confirmed with longitudinal data or a clinical trial, it only provides information about which items are linked but cannot differentiate whether item A influences item B or vice-versa, Dablander and Hinne^19^ call this the problem of items being a “root or leaf” node. Therefore, network analysis is a useful tool to map out the bidirectional connections between symptoms and to generate an understanding of which items and sub-scales are related, but the hypotheses it may generate should be followed-up with confirmatory methods. Also effects of different language versions of the PDQ-39 might cause differences in network patterns between different cohorts. The used samples are not fully representative of the PD population due to inclusion and exclusion criteria (i.e., age limit, no dementia, no severe comorbidities, etc.). In addition, the estimation of a stable network usually requires larger sample sizes and this limits its applicability to smaller local cohorts. Network analysis remains an explorative approach, but—as for all explorative methods—confirmatory analysis methods are available (e.g., to test if targeting central PDQ-39 symptoms truly has a large downstream effect on overall QoL).

## Conclusion

In two independent study cohorts of PD patients, network analysis identified depressive mood, feeling isolated, feeling embarrassed, and having trouble getting around in public/needing company when going out as well-connected items of the PDQ-39, meaning that these items are closely intertwined with other PDQ-39 items. Our results thus help understand how different aspects of QoL are linked in PD patients. Additionally, we confirm previous concerns regarding the validity of the PDQ-39 subscales, especially the SOC, COM and BOD and COG subscales. These results highlight the need to use the PDQ-39 subscales with caution as outcome measure for health-related QoL in studies with PD patients.

## Methods

### Samples

We used data from two independent samples of PD patients without PD dementia:

Data from the COhort of Patients with PArkinson's DIsease in Spain (COPPADIS) study was used to estimate the PDQ-39 network. In the COPPADIS study, PD patients were recruited from 35 centers in Spain between January 2016 and November 2017 (N = 694 patients)^23^. The descriptive statistic of the cohort is given in Supplement Table 1. Distributions of PDQ-39 on the item and scale level are given in Supplement Table 4.

Additionally, we used data from a Chinese observational study of 283 people with PD recruited from the Department of Neurology of Huashan hospital affiliated with Fudan University, Shanghai, China, between March and August 2012^15^. The descriptive statistic of the cohort is given in Supplement Table 1.

### Ethics approval

Data collection for the COPPADIS study was approved by the respective ethics committees of each recruiting center (45 centers in Spain, e.g. Hospitalario Universitario de Ferrol, A Coruña, see Santos-García et al. (2015)^17^ for the full list); the study by Chen et al. was approved by the ethics committee of Huashan Hospital, Shanghai. Data collection was performed according to the Declaration of Helsinki and all included participants provided written informed consent^15,23^.

### Statistical analyses

All analyses were conducted using IBM SPSS statistics (Version 25), JASP (Version 0.16), and R (Version 4.1.1), especially the R packages *bootnet* (Version 1.5)^10^ and *qgraph* (Version 1.9)^24^. For a detailed description of network analysis in R, see Costantini et al.^25^ and Epskamp et al.^10^. Descriptive statistics were used to initially describe the cohorts.

As described above, networks contain two fundamental components: *nodes*, meaning the variables entered into the network model, and *edges*. Edges describe the relationship between two nodes. Here, we computed an undirected and weighted network model. The strength of the relationship between nodes is indicated in terms of edge weights, with blue edges indicating positive relations and red edges indicating negative relations. Edge weights (i.e., strength of the relation between nodes) are reflected by the thickness of the edge, with thicker lines indicating stronger relationships^24^. We used a network approach based on the Gaussian Graphical Model as introduced by Lauritzen^26^, in which each edge represents the partial correlation coefficient between two nodes while conditioning on the other variables in the network^10^. The nodes are positioned using the Fruchterman-Reingold algorithm, which organizes the network based on the strength of the connections between nodes. Nodes with strong similarity are positioned closer together. In a simple correlation network, all significant and non-significant edges are shown, causing a jumbled network with much noise. Therefore, instead of using simple correlations, we used a regularized estimation method called the Extended Bayesian Information Criterion Graphical Least Absolute Shrinkage and Selection Operator (EBICglasso)^10^ to estimate the partial correlations between all variables and shrink the absolute weights to zero. Hence, edge weights which were shrunken to exactly zero do not have to be tested against zero anymore, alleviating the problem of multiple testing. Consequently, the network produced a model that is sparser and easier to interpret, which is especially helpful as the number of nodes and edges in a network may influence the skewness of centrality measures^19^. As the PDQ-39 contains a high number of items, the extended Bayesian Information Criteria (EBIC) was used as an information criterion that takes both model complexity and model fit into account^27^. The hyperparameter was set at 0.5^28^. Missing data was handled using the “exclude pairwise method”. As Isvoranu and Epskamp^29^ showed in several data simulations, transformation to normalize ordered categorical data did not improve network accuracy, thus we did not perform this step and analyzed the data as is. Thus, as the PDQ-39 contains ordinal data, we

In addition to visualizing the relationships between variables, the network can also be described statistically in terms of *edge weights* and *centrality* measures. *Centrality* refers to the relative importance of a node and its connection to other nodes within the network. Of note, the centrality of a node does not equal its mean value, as it does not represent symptom severity (as perceived by the respective study participant) per se, but rather an item’s connectedness with other items in the network^17^. A node with high centrality is therefore highly connected to other nodes, and changes in this node can be expected to quickly spread to other nodes. Nodes with low centrality have less influence on the network. As a debate exists on the applicability of centrality indices for psychometric or medical data^19,21,22^, we decided to include only *strength* and *expected influence* as indicators of centrality. These are the most suitable for our type of data, especially when paired with an EBICglasso approach and bootstrapping to assess their stability^28–30^.

The *strength* (also called *degree* in unweighted networks) is the sum of all direct associations a node has with other nodes. It reflects the bundled information flow of a node in the network, although in undirected networks it is not clear whether this strength is rooted in the variable being a predictor for other variables, or a consequence of other items^19^. Clinically, a node with a high strength has the potential to represent an important feature (for example as an item of a psychological construct), because a change in the value of this node has a strong direct and quick effect on other nodes within the network. We used a bootstrapping approach to identify significant differences in the strength centrality measure of the variables within each network^10^.

To identify highly influential nodes more reliably, it may be necessary to distinguish between positive and negative edges and therefore the *expected influence* centrality measures was developed^14^. The expected influence therefore assesses the node´s cumulative influence within the network.

Finally, measures of network accuracy and stability were calculated. A case-dropping bootstrap procedure was used to examine the stability of node strength. This approach assumes that a network is stable if a large proportion of the sample can be excluded from the dataset without observing significant changes in the indices. For a stable network, the Correlation Stability Coefficient (CS-C) should lie above 0.5^10^. A nonparametric bootstrap procedure based on 95% confidence intervals (CIs) was used to assess the edge weights stability, with narrow 95% CIs indicating a more trustworthy network^10^. Bootstrap stability difference test was used to test for statistical significance both between edge-weights that were non-zero and between nodes in terms of strength centrality^10^.

Based on the findings from network analyses, confirmatory factor analyses (CFA) using JASP (Version 0.16) were conducted with a diagonally weighted least square (DWLS) estimator. Goodness-of-fit was evaluated using the χ^2^ statistic of exact fit, comparative fit index (CFI), Tucker–Lewis index (TLI), and root mean square error of approximation (RMSEA). TLI and CFI should be > 0.90, and RMSEA < 0.08 for an acceptable fit. As no universally agreed-upon cut-off is defined for appropriate factor loadings—some researchers suggest a cut-off of 0.4^31,32^, others define a cut-off of 0.6^33,34^—we did not define a cut-off for factor loadings in our analysis. Instead, we focus on overall model fit as described above and interpret the associations between items and their respective factors in line with the context and the results from network analysis. As QoL is a highly complex construct and the PDQ-39 contains a multitude of symptoms that may influence it, some variation in factor loadings is to be expected.

Of note, the inclusion of traditional factor analysis methods and network analysis is beneficial as both contribute valuable information that may be concordant but nonetheless differs in its interpretation. Unlike factor analysis, where a data structure is estimated to assess an underlying latent variable that the included data aims to explain, network analysis considers the relation between different items and how they are associated with each other, this includes a potential display of information flow from one item to another via a third item. Thereby, network analysis can furthermore map out the associations between different sub-scales of a questionnaire and identify bridging variables that connect different aspects of an illness^12,16^.

## Supplementary Information

Supplementary Information.

## Data Availability

Data underlying the analyses are available from the corresponding authors from the respective studies^15,23^.
