# Supplementary material for: Using network analysis to explore the validity and influential items of the Parkinson’s Disease Questionnaire-39
Source: Sci Rep. 2023 May 3;13:7221. doi: 10.1038/s41598-023-34412-4 (PMC10156662; doi:10.1038/s41598-023-34412-4)
Supplement: Supplementary file 1 — Supplementary Information. [file 41598_2023_34412_MOESM1_ESM.docx]

**Supplementary Materials for *Using network analysis to explore the validity and influential items of the Parkinson’s Disease Questionnaire-39***

[Supplement Figure 1. Strength stability for COPPADIS cohort network based on 1,000 case-drop bootstraps 2](#_Toc119485128)

[Supplement Figure 2. Bootstrapped 95% confidence intervals of edge weights for COPPADIS cohort network 3](#_Toc119485129)

[Supplement Figure 3. Centrality difference test, based on 1,000 parametric bootstrap samples for COPPADIS cohort network 4](#_Toc119485130)

[Supplement Figure 4. Network plot for data from Chen et al. 2017 (A) and COPPADIS (B) 5](#_Toc119485131)

[Supplement Figure 5. Centrality measures for data from COPPADIS (green lines) and Chen et al. 2017 (orange lines)(normalized values) 7](#_Toc119485132)

[Supplement Figure 6. Strength stability for cohort network by Chen et al. 2017 based on 1,000 case-drop bootstraps 8](#_Toc119485133)

[Supplement Figure 7. Bootstrapped 95% confidence intervals of edge weights for cohort network by Chen et al. 2017 9](#_Toc119485134)

[Supplement Figure 8. Centrality difference test, based on 1,000 parametric bootstrap samples for cohort network by Chen et al. 2017 10](#_Toc119485135)

[Supplement Table 1. Descriptive statistics with group differences 11](#_Toc119485136)

[Supplement Table 2. Centrality measures per variable normalized (COPPADIS) 12](#_Toc119485137)

[Supplement Table 3. Weights matrix 13](#_Toc119485138)

[Supplement Table 4. Detailed descriptive statistics on item and scale PDQ-39 level (COPPADIS study, N = 694) 15](#_Toc119485139)

[Supplement Table 5. Confirmatory factor analysis of PDQ-39 items (items arranged according to original description be Peto et al. 1995) with data from COPPADIS study 17](#_Toc119485140)

[Supplement Table 6. Confirmatory factor analysis of PDQ-39 items (items arranged according to original description be Peto et al. 1995, but “ignored” categorized into SOC) with data from COPPADIS study 19](#_Toc119485141)

[Supplement Table 7. Confirmatory factor analysis of PDQ-39 items (items arranged according to original description be Peto et al. 1995) with data from Chen at al. 2017 21](#_Toc119485142)

[Supplement Table 8. Confirmatory factor analysis of PDQ-39 items (items arranged according to original description be Peto et al. 1995, but “ignored” categorized into SOC) with data from Chen et al. 2017 23](#_Toc119485143)

[References 25](#_Toc119485144)

# Supplement Figure 1. Strength stability for COPPADIS cohort network based on 1,000 case-drop bootstraps


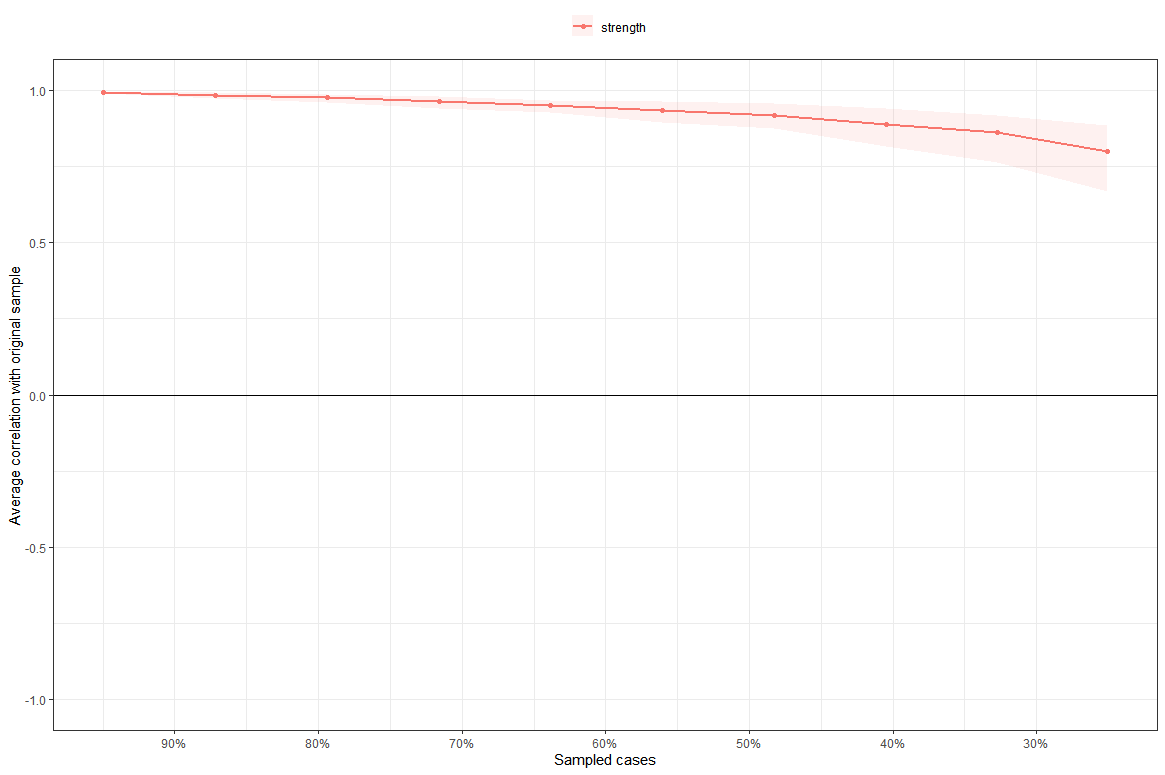


Note: To investigate the stability of the strength centrality index, a case-dropping bootstrap procedure was used^1^. This re-estimates strength with an increasingly higher percentage of dropped-out cases. Then the correlation between the original strength index and those from the subsamples were calculated.

# Supplement Figure 2. Bootstrapped 95% confidence intervals of edge weights for COPPADIS cohort network

**
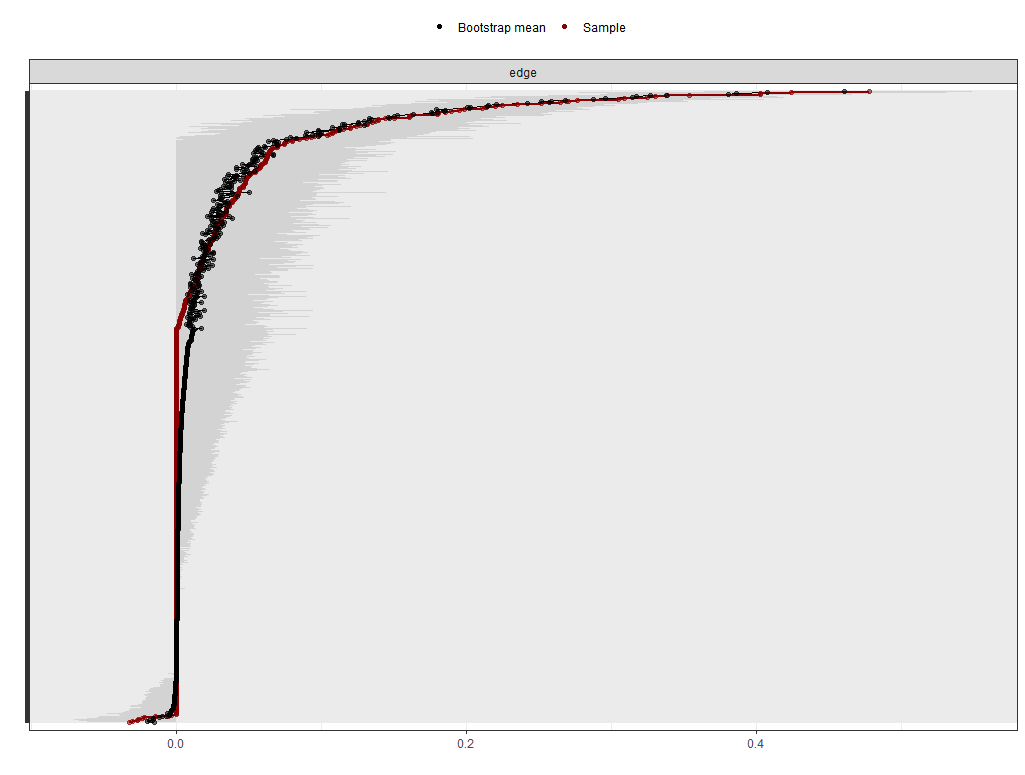
**

Note: Bootstrapped 95% confidence intervals of edge weights, based on 1,000 nonparametric bootstrap samples: A nonparametric bootstrap method and construction of 95% bootstrapped confidence interval around the regularized edge weight were used to investigate the stability of edge weight parameters. The red dots represent the original sample values. The black dots represent the bootstrap means, and the grey areas represent the 95% bootstrapped confidence interval. The bootstrapped confidence interval is very narrow, suggesting highly stable and interpretable results.

# Supplement Figure 3. Centrality difference test, based on 1,000 parametric bootstrap samples for COPPADIS cohort network


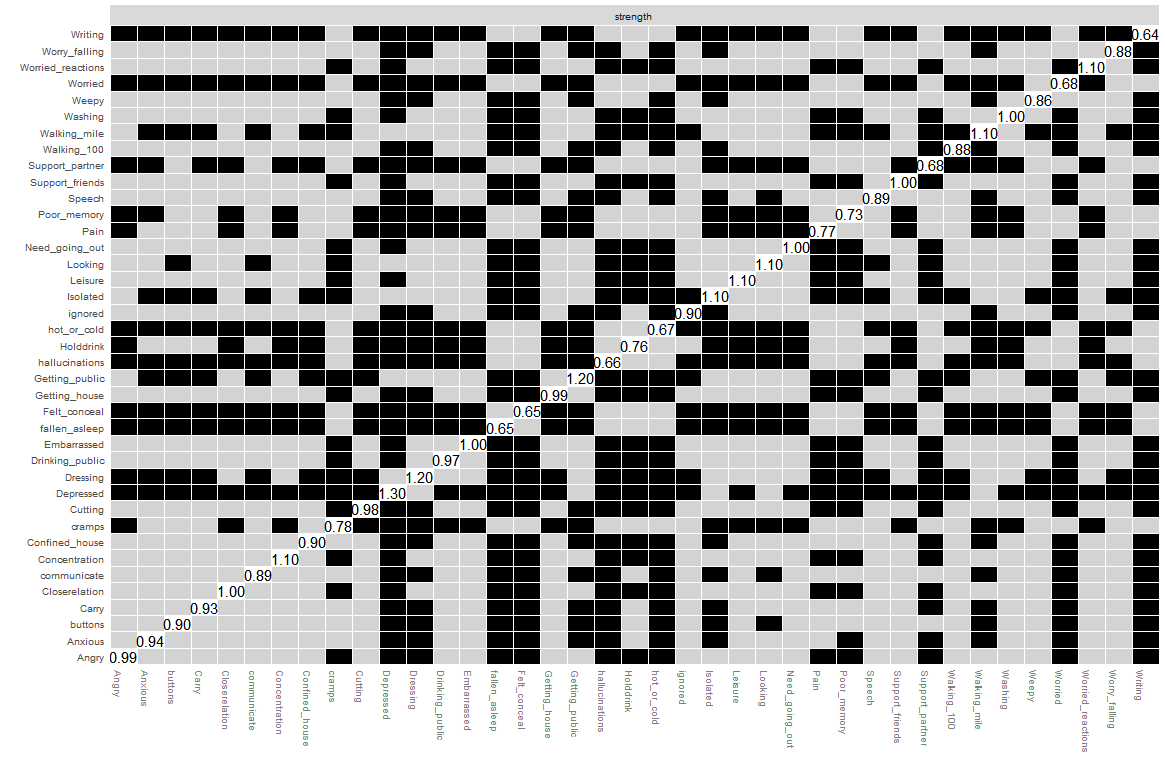


Note: A bootstrap stability difference test (á = 0.05) was used to explore if the nodes were significantly different than each other in terms of strength centrality. Each point on the x and y axes represents a network node. Gray boxes indicate that two nodes do not significantly differ from each other. Black boxes indicate that two nodes significantly differ from each other. The numbers in the diagonal represent the values of the strength centrality measure of the node.

Abbreviations: Leisure (Leisure activities), Looking (Looking after home), Carry (Carry shopping bags), Walking_mile (Walking half a mile), Walking_100 (Walking 100 yards), Getting_house (Getting around the house), Getting_public (Getting around in public), Need_going_out (Need company when going out), Worry_falling (Worry falling in public), Confined_house (Confined to the house), Washing (Washing), Dressing (Dressing), buttons (Do buttons or shoe laces), Writing (Writing clearly), Cutting (Cutting food), Holddrink (Hold a drink without spilling), Depressed (Depressed), Isolated (Isolated and lonely), Weepy (Weepy or tearful), Angry (Angry or bitter), Anxious (Anxious), Worried (Worried about the future), Felt_conceal (Felt need to conceal PD), Drinking_public (Avoid eating/drinking in public), Embarrassed (Embarrassed due to PD), Worried_reactions (Worried people's reactions), Closerelation (Close relationships), Support_partner (Support from partner), Support_friends (Support from family or friends), fallen_asleep (Unexpectedly fallen asleep), Concentration (Concentration), Poor_memory (Poor memory), hallucinations (Dreams or hallucinations), Speech (Speech), communicate (Unable communicate properly), ignored (Felt ignored), cramps (Painful cramps or spasms), Pain (Pain in joints or body), hot_or_cold (Unpleasantly hot or cold).

# Supplement Figure 4. Network plot for data from Chen et al. 2017 (A) and COPPADIS (B)

**
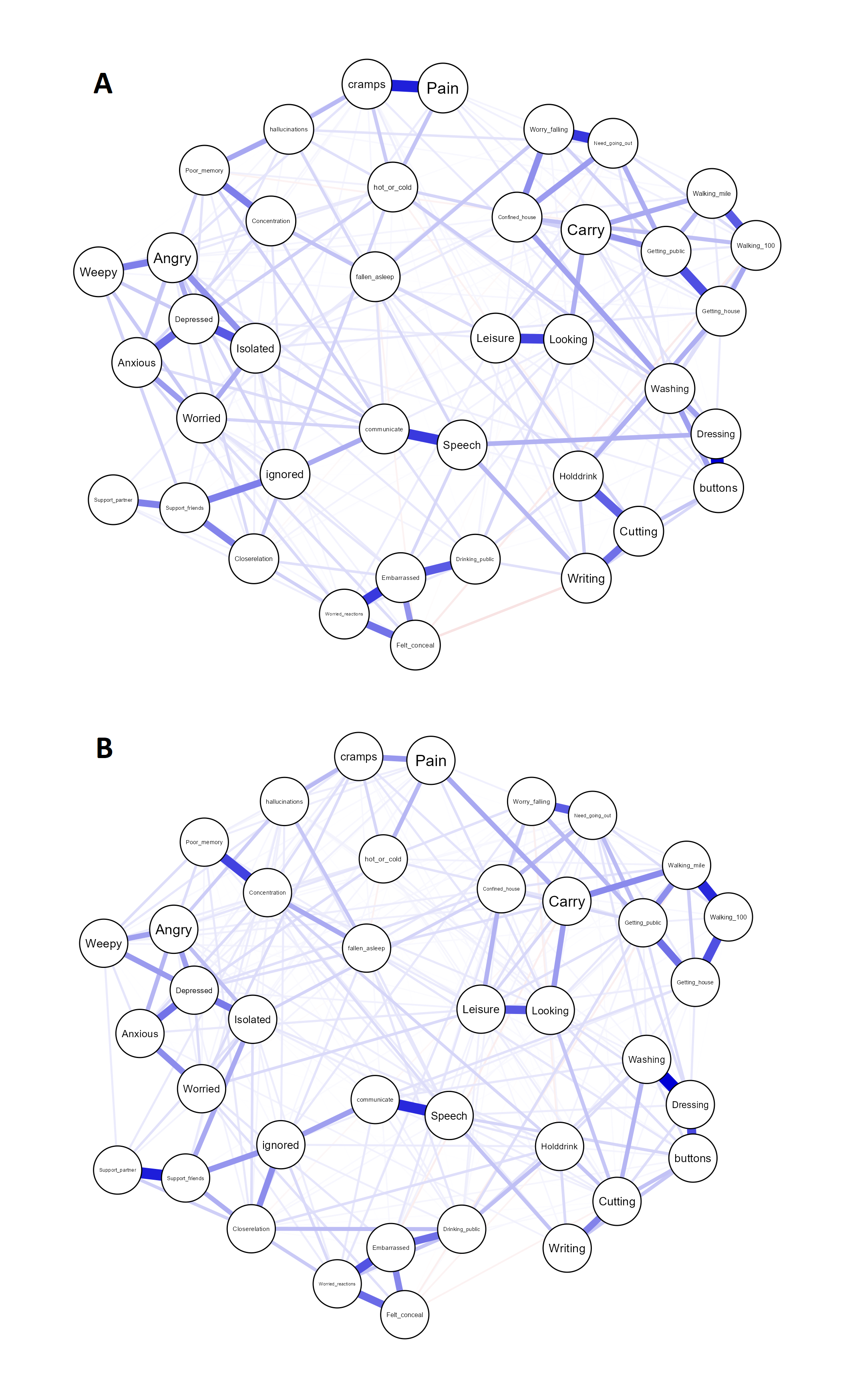
**

**
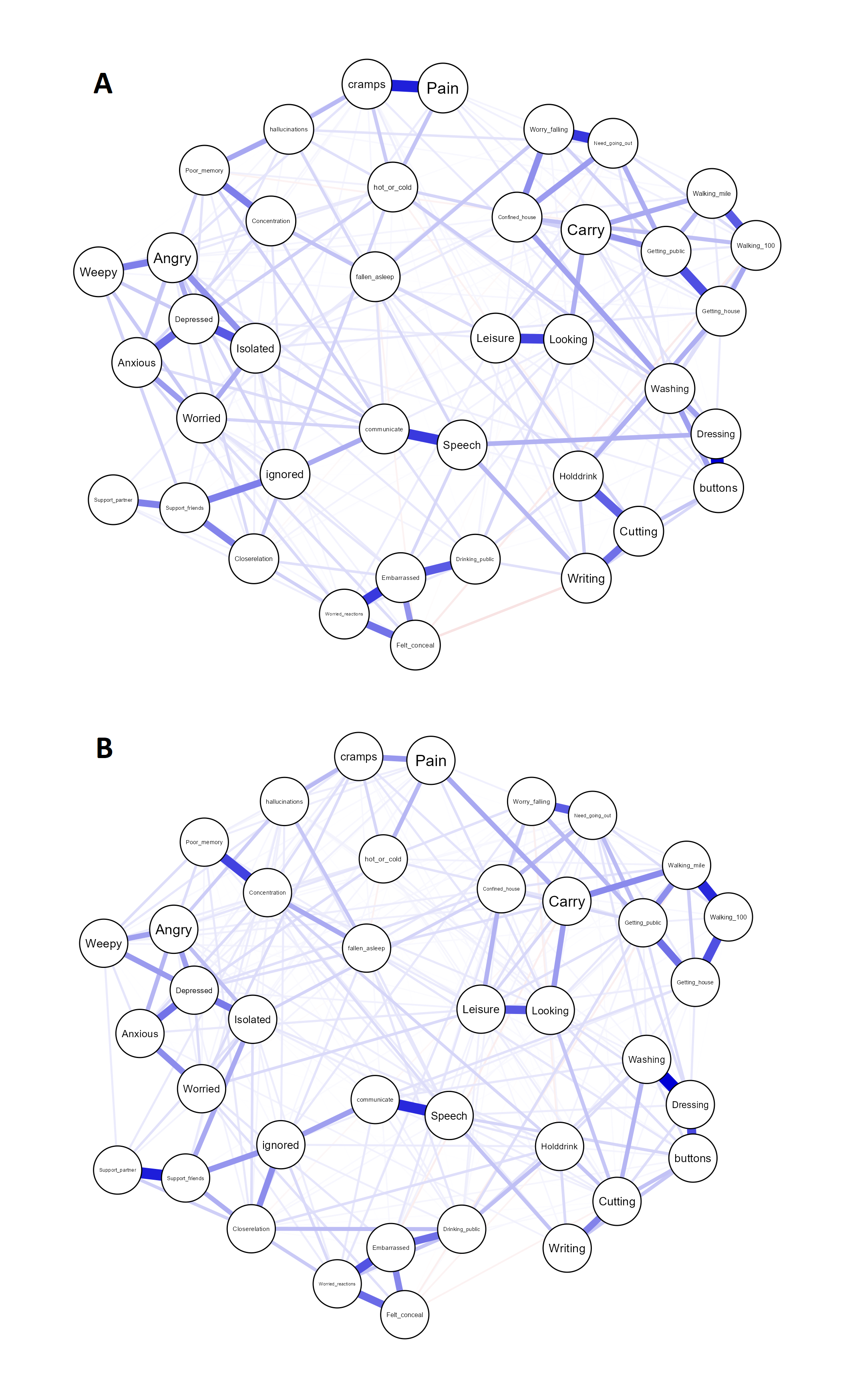
**

# Supplement Figure 5. Centrality measures for data from COPPADIS (green lines) and Chen et al. 2017 (orange lines)(normalized values)

**
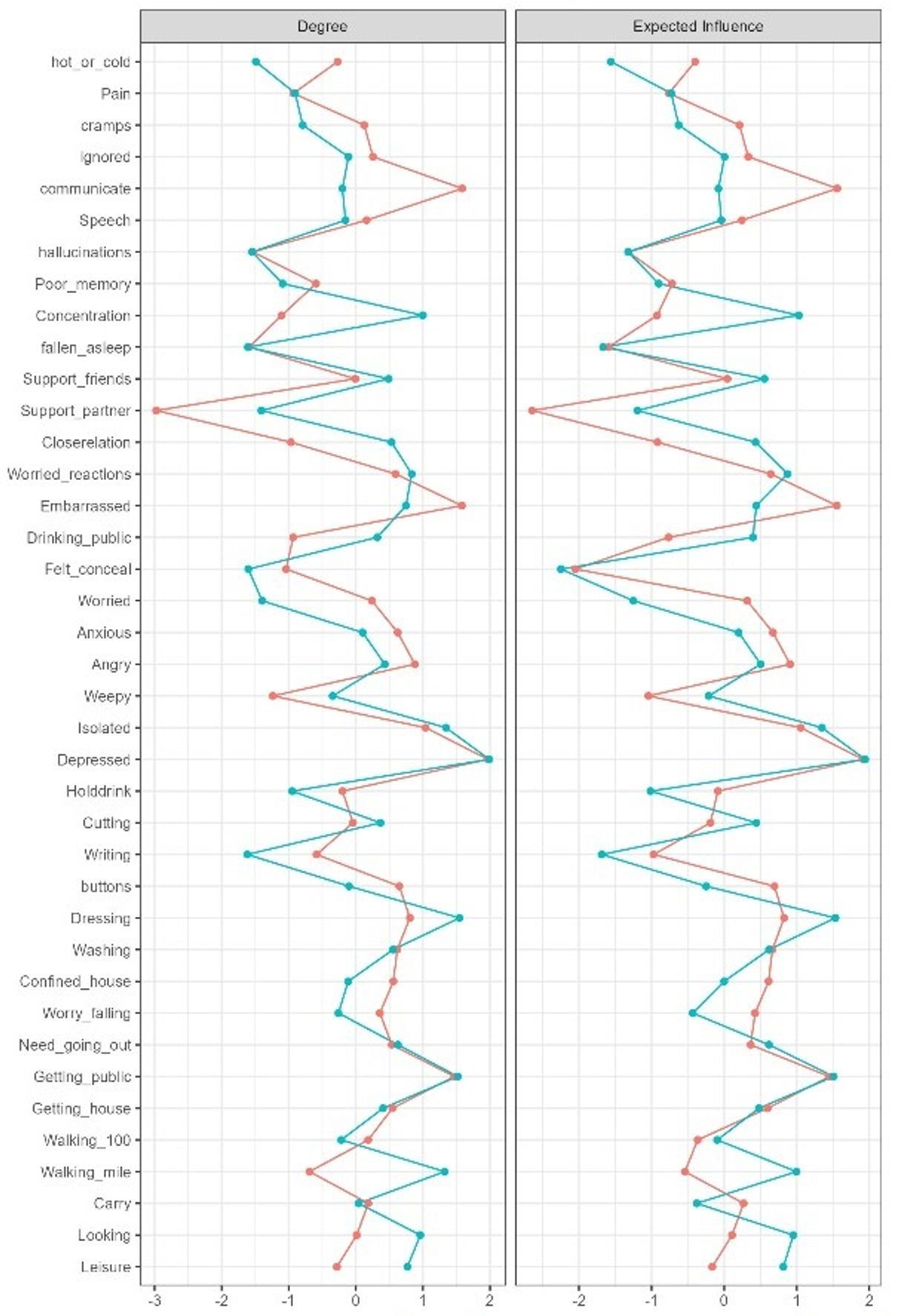
**

# Supplement Figure 6. Strength stability for cohort network by Chen et al. 2017 based on 1,000 case-drop bootstraps


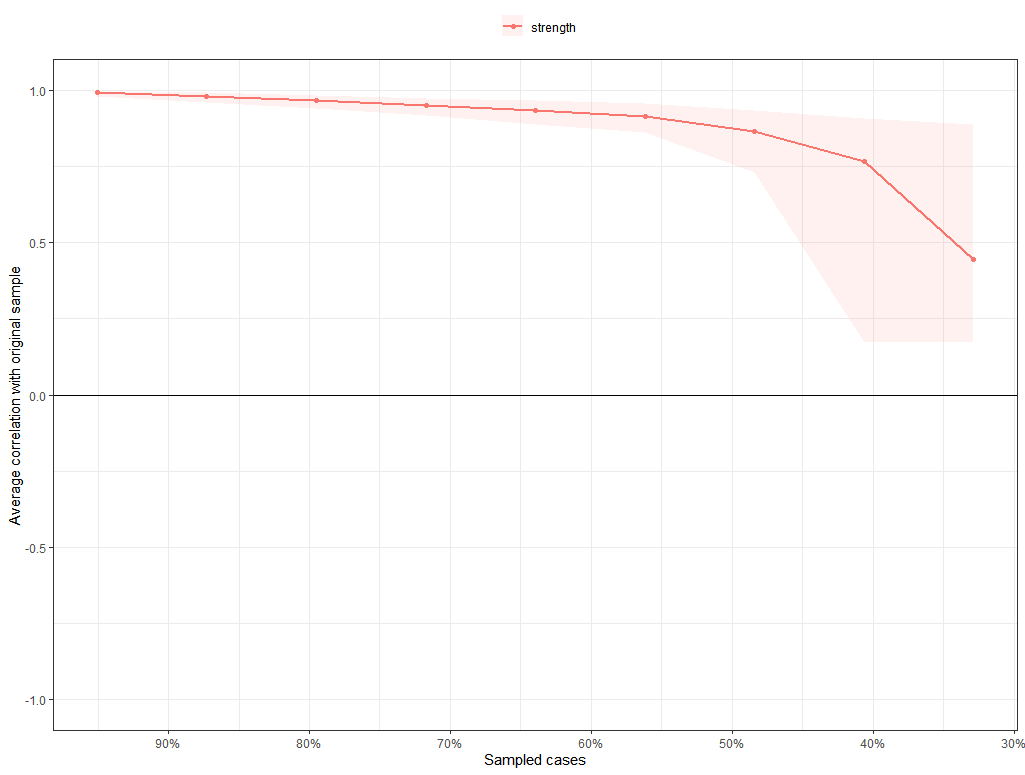


Note: To investigate the stability of the strength centrality index, a case-dropping bootstrap procedure was used ^1^. This re-estimates strength with an increasingly higher percentage of dropped-out cases. Then the correlation between the original strength index and those from the subsamples were calculated.

# Supplement Figure 7. Bootstrapped 95% confidence intervals of edge weights for cohort network by Chen et al. 2017

**
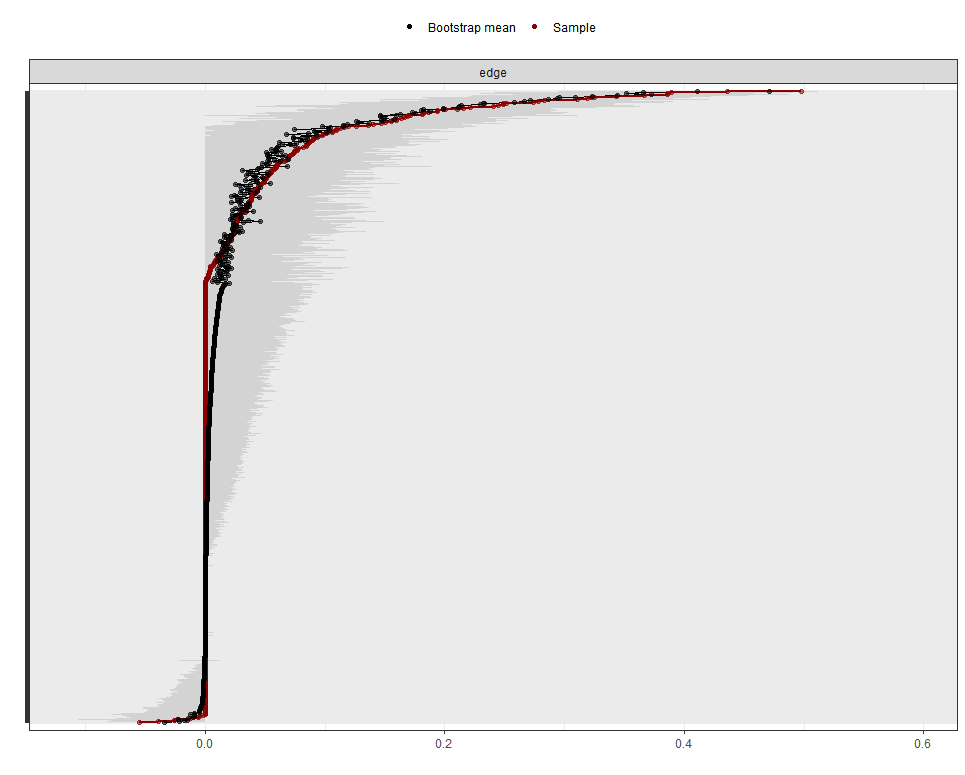
**

Note: Bootstrapped 95% confidence intervals of edge weights, based on 1,000 nonparametric bootstrap samples: A nonparametric bootstrap method and construction of 95% bootstrapped confidence interval around the regularized edge weight were used to investigate the stability of edge weight parameters. The red dots represent the original sample values. The black dots represent the bootstrap means, and the grey areas represent the 95% bootstrapped confidence interval. The bootstrapped confidence interval is very narrow, suggesting highly stable and interpretable results.

# Supplement Figure 8. Centrality difference test, based on 1,000 parametric bootstrap samples for cohort network by Chen et al. 2017


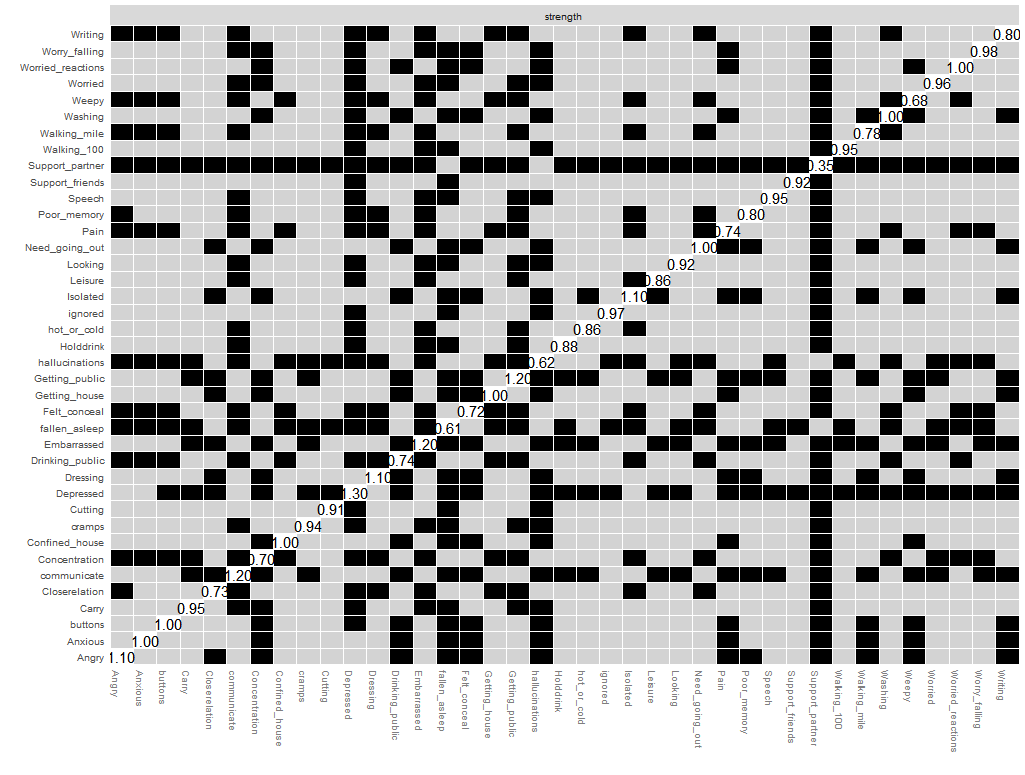


Note: A bootstrap stability difference test (á = 0.05) was used to explore if the nodes were significantly different than each other in terms of strength centrality. Each point on the x and y axes represents a network node. Gray boxes indicate that two nodes do not significantly differ from each other. Black boxes indicate that two nodes significantly differ from each other. The numbers in the diagonal represent the values of the strength centrality measure of the node.

Abbreviations: Leisure (Leisure activities), Looking (Looking after home), Carry (Carry shopping bags), Walking_mile (Walking half a mile), Walking_100 (Walking 100 yards), Getting_house (Getting around the house), Getting_public (Getting around in public), Need_going_out (Need company when going out), Worry_falling (Worry falling in public), Confined_house (Confined to the house), Washing (Washing), Dressing (Dressing), buttons (Do buttons or shoe laces), Writing (Writing clearly), Cutting (Cutting food), Holddrink (Hold a drink without spilling), Depressed (Depressed), Isolated (Isolated and lonely), Weepy (Weepy or tearful), Angry (Angry or bitter), Anxious (Anxious), Worried (Worried about the future), Felt_conceal (Felt need to conceal PD), Drinking_public (Avoid eating/drinking in public), Embarrassed (Embarrassed due to PD), Worried_reactions (Worried people's reactions), Closerelation (Close relationships), Support_partner (Support from partner), Support_friends (Support from family or friends), fallen_asleep (Unexpectedly fallen asleep), Concentration (Concentration), Poor_memory (Poor memory), hallucinations (Dreams or hallucinations), Speech (Speech), communicate (Unable communicate properly), ignored (Felt ignored), cramps (Painful cramps or spasms), Pain (Pain in joints or body), hot_or_cold (Unpleasantly hot or cold).

# Supplement Table 1. Descriptive statistics with group differences

|  |  | **Chen et al. 2017** | | | | **COPPADIS** | | | |
| --- | --- | --- | --- | --- | --- | --- | --- | --- | --- |
|  |  | **n** | **%** |  | | **n** | **%** |  | |
| Sex | female | 117 | 41.3 |  |  | 276 | 39.8 |  |  |
|  | male | 166 | 58.7 |  |  | 418 | 60.2 |  |  |
| Hoehn & Yahr stage (off) | Missing | 0 | 0.0 |  |  | 72 | 7.4 |  |  |
|  | 1 | 70 | 7.2 |  |  | 87 | 8.9 |  |  |
|  | 1.5 | 1 | 0.1 |  |  | 54 | 5.5 |  |  |
|  | 2 | 125 | 12.8 |  |  | 365 | 37.4 |  |  |
|  | 2.5 | 5 | 0.5 |  |  | 58 | 5.9 |  |  |
|  | 3 | 73 | 7.5 |  |  | 4 | 5.0 |  |  |
|  | 4 | 7 | 0.7 |  |  | 9_a_ | 0.9 |  |  |
|  | 5 | 2 | 0.2 |  |  | 0 | 0.0 |  |  |
|  | | **M** | **SD** | **95%CI lower** | **95%CI upper** | **M** | **SD** | **95%CI lower** | **95%CI upper** |
| Age | | *54.48_a_* | *12.36* | *53.03* | *55.93* | *62.59_b_* | *8.92* | *61.93* | *63.26* |
| UPDRSIII_OFF | | *30.27_a_* | *13.68* | *28.67* | *31.87* | *22.68_b_* | *11.18* | *21.80* | *23.55* |
| LEDD | | 249.12^1^ | 261.27 | 214.24 | 284.00 | NA | . | . | . |
| BDI | | *12.76_a_* | *8.80* | *11.73* | *13.79* | *8.74_b_* | *7.32* | *8.19* | *9.29* |
| MMSE | | *27.82_a_* | *1.98* | *27.58* | *28.05* | *29.19_b_* | *1.05* | *29.12* | *29.27* |
| BOD | | *20.50_a_* | *20.27* | *18.13* | *22.87* | *16.62_b_* | *19.21* | *15.19* | *18.05* |
| MOB | | 16.92_a_ | 18.84 | 14.71 | 19.12 | 18.02_a_ | 18.62 | 16.63 | 19.41 |
| ADL | | 23.20_a_ | 20.36 | 20.82 | 25.59 | 21.49_a_ | 19.97 | 20.01 | 22.98 |
| EMO | | *24.38_a_* | *24.63* | *21.50* | *27.26* | *13.44_b_* | *19.47* | *11.99* | *14.89* |
| STI | | *12.69_a_* | *24.53* | *9.82* | *15.56* | *8.24_b_* | *16.54* | *7.00* | *9.47* |
| SOC | | *23.50_a_* | *18.16* | *21.37* | *25.62* | *19.30_b_* | *17.86* | *17.97* | *20.63* |
| COG | | *13.52_a_* | *18.21* | *11.39* | *15.65* | *10.22_b_* | *15.28* | *9.08* | *11.36* |
| COM | | 27.74_a_ | 23.30 | 25.01 | 30.46 | 26.40_a_ | 22.83 | 24.70 | 28.11 |
| PDQ-39 Summary index | | *20.31_a_* | *14.54* | *18.61* | *22.01* | *16.72_b_* | *12.82* | *15.76* | *17.67* |
| Note: Cursive values in the same row and sub-table where the subscript is not identical differ strongly at p <.05 in the two-tailed test (t-test, Chi-square test).  Abbreviations: Unified Parkinson's Disease Rating Scale (UPDRS), levodopa equivalent daily dose (LEDD), Beck´s depression inventory (BDI), Mini Mental Status Examination (MMSE), mobility (MOB), activities of daily living (ADL), emotional well-being (EMO), stigma (STI), social support (SOC), cognition (COG), communication (COM), and bodily discomfort (BOD), not applicable (NA). | | | | | | | | | |

# Supplement Table 2. Centrality measures per variable (COPPADIS)

| **Variable** | **Strength** | **Expected influence** |
| --- | --- | --- |
| Leisure | 0.088 | 0.252 |
| Looking | 0.486 | 0.455 |
| Carry | -0.083 | -0.382 |
| Walking_mile | 1.172 | 0.780 |
| Walking_100 | 0.286 | 0.427 |
| Getting_house | 0.143 | 0.300 |
| Getting_public | 0.822 | 0.900 |
| Need_going_out | 1.751 | 1.564 |
| Worry_falling | -0.418 | -0.544 |
| Confined_house | -0.293 | -0.084 |
| Washing | 0.742 | 0.829 |
| Dressing | 1.540 | 1.534 |
| buttons | -0.487 | -0.702 |
| Writing | -1.762 | -1.731 |
| Cutting | 0.177 | 0.330 |
| Holddrink | -0.768 | -1.107 |
| Depressed | 1.528 | 1.524 |
| Isolated | 1.312 | 1.333 |
| Weepy | -0.673 | -0.576 |
| Angry | 0.300 | 0.439 |
| Anxious | -0.398 | -0.177 |
| Worried | -1.750 | -1.528 |
| Felt_conceal | -0.502 | -2.131 |
| Drinking_public | 0.805 | 0.885 |
| Embarrassed | 1.317 | 0.822 |
| Worried_reactions | 0.596 | 0.700 |
| Closerelation | 0.767 | 0.810 |
| Support_partner | -0.926 | -0.644 |
| Support_friends | 1.144 | 1.185 |
| fallen_asleep | -1.836 | -1.804 |
| Concentration | 0.666 | 0.635 |
| Poor_memory | -1.386 | -1.050 |
| hallucinations | -1.186 | -0.873 |
| Speech | -0.439 | -0.214 |
| communicate | 0.422 | 0.547 |
| ignored | 0.371 | 0.388 |
| cramps | -1.059 | -0.762 |
| Pain | -1.302 | -1.118 |
| hot_or_cold | -1.166 | -1.213 |
| Abbreviations: Leisure (Leisure activities), Looking (Looking after home), Carry (Carry shopping bags), Walking_mile (Walking half a mile), Walking_100 (Walking 100 yards), Getting_house (Getting around the house), Getting_public (Getting around in public), Need_going_out (Need company when going out), Worry_falling (Worry falling in public), Confined_house (Confined to the house), Washing (Washing), Dressing (Dressing), buttons (Do buttons or shoe laces), Writing (Writing clearly), Cutting (Cutting food), Holddrink (Hold a drink without spilling), Depressed (Depressed), Isolated (Isolated and lonely), Weepy (Weepy or tearful), Angry (Angry or bitter), Anxious (Anxious), Worried (Worried about the future), Felt_conceal (Felt need to conceal PD), Drinking_public (Avoid eating/drinking in public), Embarrassed (Embarrassed due to PD), Worried_reactions (Worried people's reactions), Closerelation (Close relationships), Support_partner (Support from partner), Support_friends (Support from family or friends), fallen_asleep (Unexpectedly fallen asleep), Concentration (Concentration), Poor_memory (Poor memory), hallucinations (Dreams or hallucinations), Speech (Speech), communicate (Unable communicate properly), ignored (Felt ignored), cramps (Painful cramps or spasms), Pain (Pain in joints or body), hot_or_cold (Unpleasantly hot or cold). | | |

| **Variable** | **1** | **2** | **3** | **4** | **5** | **6** | **7** | **8** | **9** | **10** | **11** | **12** | **13** | **14** | **15** | **16** | **17** | **18** | **19** | **20** | **21** | **22** | **23** | **24** | **25** | **26** | **27** | **28** | **29** | **30** | **31** | **32** | **33** | **34** | **35** | **36** | **37** | **38** | **39** |
| --- | --- | --- | --- | --- | --- | --- | --- | --- | --- | --- | --- | --- | --- | --- | --- | --- | --- | --- | --- | --- | --- | --- | --- | --- | --- | --- | --- | --- | --- | --- | --- | --- | --- | --- | --- | --- | --- | --- | --- |
|  | **Leisure** | **Looking** | **Carry** | **Walking_mile** | **Walking_100** | **Getting_house** | **Getting_public** | **Need_going_out** | **Worry_falling** | **Confined_house** | **Washing** | **Dressing** | **buttons** | **Writing** | **Cutting** | **Holddrink** | **Depressed** | **Isolated** | **Weepy** | **Angry** | **Anxious** | **Worried** | **Felt_conceal** | **Drinking_public** | **Embarrassed** | **Worried_reactions** | **Closerelation** | **Support_partner** | **Support_friends** | **fallen_asleep** | **Concentration** | **Poor_memory** | **hallucinations** | **Speech** | **communicate** | **ignored** | **cramps** | **Pain** | **hot_or_cold** |
| **1** | 0 | .328 | .067 | 0 | 0 | .029 | .064 | .064 | 0 | .168 | 0 | 0 | 0 | .008 | .028 | 0 | .002 | 0 | 0 | 0 | .039 | .086 | 0 | .018 | .015 | 0 | 0 | 0 | 0 | .011 | 0 | .02 | 0 | .019 | 0 | 0 | 0 | 0 | 0 |
| **2** | .328 | 0 | .194 | .021 | 0 | 0 | .022 | .052 | 0 | .01 | .031 | .074 | .023 | 0 | .108 | 0 | 0 | 0 | 0 | 0 | 0 | 0 | -0 | 0 | 0 | 0 | 0 | 0 | 0 | 0 | .033 | 0 | 0 | 0 | 0 | -.01 | .037 | .052 | .04 |
| **3** | .067 | .194 | 0 | .241 | 0 | 0 | .028 | .048 | .014 | .012 | 0 | 0 | 0 | 0 | 0 | 0 | .052 | 0 | 0 | 0 | 0 | 0 | 0 | 0 | -.05 | 0 | 0 | 0 | 0 | 0 | 0 | 0 | 0 | 0 | 0 | 0 | 0 | .154 | .079 |
| **4** | 0 | .021 | .241 | 0 | .47 | .105 | .186 | .022 | 0 | .048 | 0 | 0 | 0 | 0 | 0 | 0 | 0 | 0 | 0 | 0 | 0 | 0 | -.04 | 0 | 0 | 0 | 0 | 0 | 0 | 0 | 0 | 0 | 0 | 0 | 0 | 0 | 0 | .013 | .012 |
| **5** | 0 | 0 | 0 | .47 | 0 | .332 | .025 | .079 | 0 | .028 | .017 | 0 | 0 | 0 | 0 | 0 | 0 | 0 | 0 | 0 | 0 | 0 | 0 | 0 | 0 | 0 | .012 | 0 | .007 | 0 | 0 | 0 | .033 | 0 | .002 | 0 | 0 | 0 | 0 |
| **6** | .029 | 0 | 0 | .105 | .332 | 0 | .287 | .054 | .017 | 0 | 0 | .046 | 0 | 0 | 0 | .03 | 0 | 0 | 0 | 0 | 0 | 0 | 0 | 0 | 0 | 0 | 0 | 0 | 0 | 0 | 0 | 0 | 0 | .005 | .02 | .054 | 0 | 0 | 0 |
| **7** | .064 | .022 | .028 | .186 | .025 | .287 | 0 | .153 | .109 | .052 | .033 | .043 | 0 | 0 | 0 | 0 | 0 | 0 | 0 | 0 | 0 | 0 | 0 | .085 | 0 | 0 | 0 | 0 | 0 | 0 | 0 | 0 | 0 | 0 | .013 | 0 | 0 | 0 | 0 |
| **8** | .064 | .052 | .048 | .022 | .079 | .054 | .153 | 0 | .365 | .137 | .02 | .076 | 0 | 0 | .022 | 0 | .017 | 0 | .045 | .014 | 0 | -.02 | 0 | 0 | .028 | 0 | 0 | 0 | 0 | .05 | 0 | 0 | 0 | 0 | .003 | 0 | 0 | 0 | 0 |
| **9** | 0 | 0 | .014 | 0 | 0 | .017 | .109 | .365 | 0 | .114 | 0 | .015 | 0 | -.04 | 0 | 0 | 0 | .006 | 0 | 0 | 0 | 0 | 0 | 0 | .043 | 0 | 0 | .025 | 0 | 0 | 0 | 0 | 0 | 0 | 0 | .032 | 0 | .022 | .081 |
| **10** | .168 | .01 | .012 | .048 | .028 | 0 | .052 | .137 | .114 | 0 | .022 | 0 | 0 | 0 | 0 | .013 | .043 | .096 | .021 | 0 | 0 | 0 | 0 | .021 | .037 | .043 | 0 | 0 | 0 | 0 | 0 | 0 | 0 | 0 | 0 | 0 | .037 | 0 | 0 |
| **11** | 0 | .031 | 0 | 0 | .017 | 0 | .033 | .02 | 0 | .022 | 0 | .487 | .102 | 0 | .164 | .071 | 0 | 0 | 0 | 0 | 0 | 0 | 0 | .049 | 0 | 0 | 0 | 0 | 0 | .02 | 0 | 0 | .028 | 0 | .041 | 0 | 0 | 0 | 0 |
| **12** | 0 | .074 | 0 | 0 | 0 | .046 | .043 | .076 | .015 | 0 | .487 | 0 | .374 | 0 | .07 | 0 | 0 | 0 | 0 | 0 | 0 | 0 | 0 | 0 | 0 | 0 | 0 | 0 | 0 | 0 | 0 | 0 | .022 | .018 | .002 | 0 | 0 | 0 | 0 |
| **13** | 0 | .023 | 0 | 0 | 0 | 0 | 0 | 0 | 0 | 0 | .102 | .374 | 0 | .102 | .09 | .003 | 0 | 0 | 0 | 0 | 0 | 0 | -.05 | 0 | 0 | 0 | 0 | 0 | 0 | .041 | 0 | 0 | 0 | .086 | 0 | 0 | 0 | 0 | 0 |
| **14** | .008 | 0 | 0 | 0 | 0 | 0 | 0 | 0 | -.04 | 0 | 0 | 0 | .102 | 0 | .288 | .072 | 0 | 0 | 0 | 0 | 0 | 0 | 0 | 0 | 0 | 0 | 0 | 0 | 0 | 0 | .026 | 0 | 0 | .107 | 0 | 0 | 0 | 0 | 0 |
| **15** | .028 | .108 | 0 | 0 | 0 | 0 | 0 | .022 | 0 | 0 | .164 | .07 | .09 | .288 | 0 | .113 | .023 | 0 | 0 | .023 | 0 | 0 | 0 | .023 | 0 | 0 | .006 | 0 | 0 | 0 | 0 | 0 | 0 | 0 | .02 | 0 | .687 | 0 | .005 |
| **16** | 0 | 0 | 0 | 0 | 0 | .03 | 0 | 0 | 0 | .013 | .071 | 0 | .003 | .072 | .113 | 0 | 0 | .034 | 0 | 0 | 0 | 0 | -.06 | .171 | .005 | 0 | .069 | 0 | 0 | 0 | .066 | 0 | .01 | 0 | .056 | .037 | 0 | 0 | .005 |
| **17** | .002 | 0 | .052 | 0 | 0 | 0 | 0 | .017 | 0 | .043 | 0 | 0 | 0 | 0 | .023 | 0 | 0 | .284 | .198 | .185 | .282 | .062 | 0 | 0 | 0 | 0 | 0 | 0 | 0 | 0 | 0 | 0 | .008 | .003 | 0 | 0 | 0 | 0 | .065 |
| **18** | 0 | 0 | 0 | 0 | 0 | 0 | 0 | 0 | .006 | .096 | 0 | 0 | 0 | 0 | 0 | .034 | .284 | 0 | .165 | .1 | 0 | .108 | 0 | 0 | 0 | .019 | .046 | .064 | .162 | 0 | 0 | .012 | .004 | 0 | 0 | .053 | 0 | .032 | 0 |
| **19** | 0 | 0 | 0 | 0 | 0 | 0 | 0 | .045 | 0 | .021 | 0 | 0 | 0 | 0 | 0 | 0 | .198 | .165 | 0 | .204 | .017 | 0 | -.02 | 0 | 0 | 0 | 0 | .022 | 0 | .007 | 0 | .051 | .003 | 0 | 0 | 0 | .049 | .011 | .023 |
| **20** | 0 | 0 | 0 | 0 | 0 | 0 | 0 | .014 | 0 | 0 | 0 | 0 | 0 | 0 | .023 | 0 | .185 | .1 | .204 | 0 | .179 | .013 | 0 | 0 | 0 | 0 | .046 | 0 | 0 | 0 | 0 | .064 | .096 | .081 | .016 | .003 | 0 | 0 | .063 |
| **21** | .039 | 0 | 0 | 0 | 0 | 0 | 0 | 0 | 0 | 0 | 0 | 0 | 0 | 0 | 0 | 0 | .282 | 0 | .017 | .179 | 0 | .212 | 0 | 0 | .03 | 0 | 0 | 0 | 0 | 0 | .074 | 0 | 0 | 0 | 0 | 0 | .036 | .013 | 0 |
| **22** | .086 | 0 | 0 | 0 | 0 | 0 | 0 | -.02 | 0 | 0 | 0 | 0 | 0 | 0 | 0 | 0 | .062 | .108 | 0 | .013 | .212 | 0 | .048 | 0 | 0 | .027 | 0 | 0 | 0 | 0 | 0 | .002 | .018 | 0 | 0 | 0 | .051 | 0 | 0 |
| **23** | 0 | -0 | 0 | -.04 | 0 | 0 | 0 | 0 | 0 | 0 | 0 | 0 | -.05 | 0 | 0 | -.06 | 0 | 0 | -.02 | 0 | 0 | .048 | 0 | .015 | .322 | .29 | 0 | 0 | 0 | 0 | -.01 | 0 | 0 | 0 | 0 | 0 | 0 | -.01 | 0 |
| **24** | .018 | 0 | 0 | 0 | 0 | 0 | .085 | 0 | 0 | .021 | .049 | 0 | 0 | 0 | .023 | .171 | 0 | 0 | 0 | 0 | 0 | 0 | .015 | 0 | .306 | .135 | .136 | 0 | 0 | .041 | .027 | 0 | 0 | 0 | .047 | .021 | 0 | 0 | 0 |
| **25** | .015 | 0 | -.05 | 0 | 0 | 0 | 0 | .028 | .043 | .037 | 0 | 0 | 0 | 0 | 0 | .005 | 0 | 0 | 0 | 0 | .03 | 0 | .322 | .306 | 0 | .316 | .024 | 0 | 0 | 0 | 0 | 0 | 0 | 0 | .008 | 0 | 0 | -0 | 0 |
| **26** | 0 | 0 | 0 | 0 | 0 | 0 | 0 | 0 | 0 | .043 | 0 | 0 | 0 | 0 | 0 | 0 | 0 | .019 | 0 | 0 | 0 | .027 | .29 | .135 | .316 | 0 | .1 | 0 | 0 | 0 | 0 | 0 | 0 | 0 | .049 | .081 | 0 | 0 | 0 |
| **27** | 0 | 0 | 0 | 0 | .012 | 0 | 0 | 0 | 0 | 0 | 0 | 0 | 0 | 0 | .006 | .069 | 0 | .046 | 0 | .046 | 0 | 0 | 0 | .136 | .024 | .1 | 0 | .126 | .166 | .068 | .029 | .034 | 0 | .006 | 0 | .217 | 0 | -0 | 0 |
| **28** | 0 | 0 | 0 | 0 | 0 | 0 | 0 | 0 | .025 | 0 | 0 | 0 | 0 | 0 | 0 | 0 | 0 | .064 | .022 | 0 | 0 | 0 | 0 | 0 | 0 | 0 | .126 | 0 | .505 | .02 | .026 | 0 | 0 | 0 | 0 | 0 | 0 | 0 | 0 |
| **29** | 0 | 0 | 0 | 0 | .007 | 0 | 0 | 0 | 0 | 0 | 0 | 0 | 0 | 0 | 0 | 0 | 0 | .162 | 0 | 0 | 0 | 0 | 0 | 0 | 0 | 0 | .166 | .505 | 0 | 0 | .013 | 0 | .102 | 0 | 0 | .202 | 0 | 0 | 0 |
| **30** | .011 | 0 | 0 | 0 | 0 | 0 | 0 | .05 | 0 | 0 | .02 | 0 | .041 | 0 | 0 | 0 | 0 | 0 | .007 | 0 | 0 | 0 | 0 | .041 | 0 | 0 | .068 | .02 | 0 | 0 | .174 | 0 | .122 | .034 | 0 | 0 | 0 | 0 | -.04 |
| **31** | 0 | .033 | 0 | 0 | 0 | 0 | 0 | 0 | 0 | 0 | 0 | 0 | 0 | .026 | 0 | .066 | 0 | 0 | 0 | 0 | .074 | 0 | -.01 | .027 | 0 | 0 | .029 | .026 | .013 | .174 | 0 | .387 | .035 | .039 | .029 | 0 | .042 | 0 | .057 |
| **32** | .02 | 0 | 0 | 0 | 0 | 0 | 0 | 0 | 0 | 0 | 0 | 0 | 0 | 0 | 0 | 0 | 0 | .012 | .051 | .064 | 0 | .002 | 0 | 0 | 0 | 0 | .034 | 0 | 0 | 0 | .387 | 0 | .022 | .023 | 0 | .025 | .029 | .031 | .006 |
| **33** | 0 | 0 | 0 | 0 | .033 | 0 | 0 | 0 | 0 | 0 | .028 | .022 | 0 | 0 | 0 | .01 | .008 | .004 | .003 | .096 | 0 | .018 | 0 | 0 | 0 | 0 | 0 | 0 | .102 | .122 | .035 | .022 | 0 | .043 | 0 | .025 | .167 | .003 | 0 |
| **34** | .019 | 0 | 0 | 0 | 0 | .005 | 0 | 0 | 0 | 0 | 0 | .018 | .086 | .107 | 0 | 0 | .003 | 0 | 0 | .081 | 0 | 0 | 0 | 0 | 0 | 0 | .006 | 0 | 0 | .034 | .039 | .023 | .043 | 0 | .491 | 0 | 0 | 0 | 0 |
| **35** | 0 | 0 | 0 | 0 | .002 | .02 | .013 | .003 | 0 | 0 | .041 | .002 | 0 | 0 | .02 | .056 | 0 | 0 | 0 | .016 | 0 | 0 | 0 | .047 | .008 | .049 | 0 | 0 | 0 | 0 | .029 | 0 | 0 | .491 | 0 | .232 | 0 | 0 | 0 |
| **36** | 0 | -.01 | 0 | 0 | 0 | .054 | 0 | 0 | .032 | 0 | 0 | 0 | 0 | 0 | 0 | .037 | 0 | .053 | 0 | .003 | 0 | 0 | 0 | .021 | 0 | .081 | .217 | 0 | .202 | 0 | 0 | .025 | .025 | 0 | .232 | 0 | 0 | 0 | .025 |
| **37** | 0 | .037 | 0 | 0 | 0 | 0 | 0 | 0 | 0 | .037 | 0 | 0 | 0 | 0 | .687 | 0 | 0 | 0 | .049 | 0 | .036 | .051 | 0 | 0 | 0 | 0 | 0 | 0 | 0 | 0 | .042 | .029 | .167 | 0 | 0 | 0 | 0 | .222 | .094 |
| **38** | 0 | .052 | .154 | .013 | 0 | 0 | 0 | 0 | .022 | 0 | 0 | 0 | 0 | 0 | 0 | 0 | 0 | .032 | .011 | 0 | .013 | 0 | -.01 | 0 | -0 | 0 | -0 | 0 | 0 | 0 | 0 | .031 | .003 | 0 | 0 | 0 | .222 | 0 | .153 |
| **39** | 0 | .04 | .079 | .012 | 0 | 0 | 0 | 0 | .081 | 0 | 0 | 0 | 0 | 0 | .005 | .005 | .065 | 0 | .023 | .063 | 0 | 0 | 0 | 0 | 0 | 0 | 0 | 0 | 0 | -.04 | .057 | .006 | 0 | 0 | 0 | .025 | .094 | .153 | 0 |

# Supplement Table 3. Weights matrix

Abbreviations for table 4: mobility (MOB), activities of daily living (ADL), emotional well-being (EMO), stigma (STI), social support (SOC), cognition (COG), communication (COM), and bodily discomfort (BOD). Leisure (Leisure activities), Looking (Looking after home), Carry (Carry shopping bags), Walking_mile (Walking half a mile), Walking_100 (Walking 100 yards), Getting_house (Getting around the house), Getting_public (Getting around in public), Need_going_out (Need company when going out), Worry_falling (Worry falling in public), Confined_house (Confined to the house), Washing (Washing), Dressing (Dressing), buttons (Do buttons or shoe laces), Writing (Writing clearly), Cutting (Cutting food), Holddrink (Hold a drink without spilling), Depressed (Depressed), Isolated (Isolated and lonely), Weepy (Weepy or tearful), Angry (Angry or bitter), Anxious (Anxious), Worried (Worried about the future), Felt_conceal (Felt need to conceal PD), Drinking_public (Avoid eating/drinking in public), Embarrassed (Embarrassed due to PD), Worried_reactions (Worried people's reactions), Closerelation (Close relationships), Support_partner (Support from partner), Support_friends (Support from family or friends), fallen_asleep (Unexpectedly fallen asleep), Concentration (Concentration), Poor_memory (Poor memory), hallucinations (Dreams or hallucinations), Speech (Speech), communicate (Unable communicate properly), ignored (Felt ignored), cramps (Painful cramps or spasms), Pain (Pain in joints or body), hot_or_cold (Unpleasantly hot or cold).

# Supplement Table 4. Detailed descriptive statistics on item and scale PDQ-39 level (COPPADIS study, N = 694)

|  | **Mean** | **SD** | **Median** | **IQR** | **Item-rest correlation** | **Cronbach´s alpha** |
| --- | --- | --- | --- | --- | --- | --- |
| MOB | 18.018 | 18.622 | 12.500 | 20.833 |  | 0.898 |
| Leisure | 1.014 | 1.199 | 1.000 | 2.000 | 0.626 |  |
| Looking | 0.996 | 1.155 | 1.000 | 2.000 | 0.630 |  |
| Carry | 1.039 | 1.351 | 0.000 | 2.000 | 0.596 |  |
| Walking_mile | 0.692 | 1.142 | 0.000 | 1.000 | 0.732 |  |
| Walking_100 | 0.363 | 0.806 | 0.000 | 0.000 | 0.655 |  |
| Getting_house | 0.441 | 0.884 | 0.000 | 1.000 | 0.680 |  |
| Getting_public | 0.635 | 1.034 | 0.000 | 1.000 | 0.759 |  |
| Need_going_out | 0.405 | 0.957 | 0.000 | 0.000 | 0.676 |  |
| Worry_falling | 0.504 | 0.983 | 0.000 | 1.000 | 0.586 |  |
| Confined_house | 0.558 | 1.019 | 0.000 | 1.000 | 0.609 |  |
| ADL | 21.494 | 19.972 | 16.667 | 29.167 |  | 0.814 |
| Washing | 0.422 | 0.871 | 0.000 | 0.000 | 0.672 |  |
| Dressing | 0.488 | 0.919 | 0.000 | 1.000 | 0.685 |  |
| buttons | 0.850 | 1.112 | 0.000 | 1.000 | 0.646 |  |
| Writing | 1.316 | 1.284 | 1.000 | 2.000 | 0.450 |  |
| Cutting | 0.725 | 1.057 | 0.000 | 1.000 | 0.647 |  |
| Holddrink | 0.523 | 0.908 | 0.000 | 1.000 | 0.435 |  |
| EMO | 13.437 | 19.472 | 0.000 | 25.000 |  |  |
| Depressed | 0.840 | 1.110 | 0.000 | 2.000 | 0.738 |  |
| Isolated | 0.507 | 0.933 | 0.000 | 1.000 | 0.641 |  |
| Weepy | 0.793 | 1.057 | 0.000 | 2.000 | 0.600 |  |
| Angry | 0.612 | 0.909 | 0.000 | 1.000 | 0.643 |  |
| Anxious | 1.099 | 1.121 | 1.000 | 2.000 | 0.635 |  |
| Worried | 1.307 | 1.271 | 1.000 | 2.000 | 0.487 |  |
| STI | 8.237 | 16.540 | 0.000 | 8.333 |  | 0.777 |
| Felt_conceal | 0.807 | 1.259 | 0.000 | 1.000 | 0.524 |  |
| Drinking_public | 0.380 | 0.849 | 0.000 | 0.000 | 0.511 |  |
| Embarrassed | 0.451 | 0.929 | 0.000 | 0.000 | 0.682 |  |
| Worried_reactions | 0.512 | 0.938 | 0.000 | 1.000 | 0.670 |  |
| SOC | 19.299 | 17.864 | 18.750 | 25.000 |  | 0.743 |
| Closerelation | 0.304 | 0.755 | 0.000 | 0.000 | 0.480 |  |
| Support_partner | 0.352 | 0.868 | 0.000 | 0.000 | 0.581 |  |
| Support_friends | 0.333 | 0.817 | 0.000 | 0.000 | 0.653 |  |
| COG | 10.219 | 15.279 | 0.000 | 16.667 |  | 0.645 |
| fallen_asleep | 0.778 | 1.110 | 0.000 | 1.000 | 0.371 |  |
| Concentration | 0.870 | 1.031 | 1.000 | 2.000 | 0.555 |  |
| Poor_memory | 1.009 | 1.074 | 1.000 | 2.000 | 0.447 |  |
| hallucinations | 0.431 | 0.878 | 0.000 | 0.000 | 0.340 |  |
| COM | 26.405 | 22.834 | 25.000 | 33.333 |  | 0.684 |
| Speech | 0.598 | 0.890 | 0.000 | 1.000 | 0.514 |  |
| communicate | 0.353 | 0.733 | 0.000 | 0.000 | 0.622 |  |
| ignored | 0.275 | 0.707 | 0.000 | 0.000 | 0.386 |  |
| BOD | 16.617 | 19.206 | 10.000 | 22.500 |  | 0.607 |
| cramps | 1.026 | 1.169 | 1.000 | 2.000 | 0.419 |  |
| Pain | 1.497 | 1.353 | 1.000 | 2.750 | 0.455 |  |
| hot_or_cold | 0.646 | 1.127 | 0.000 | 1.000 | 0.381 |  |
| Abbreviations: mobility (MOB), activities of daily living (ADL), emotional well-being (EMO), stigma (STI), social support (SOC), cognition (COG), communication (COM), and bodily discomfort (BOD). Leisure (Leisure activities), Looking (Looking after home), Carry (Carry shopping bags), Walking_mile (Walking half a mile), Walking_100 (Walking 100 yards), Getting_house (Getting around the house), Getting_public (Getting around in public), Need_going_out (Need company when going out), Worry_falling (Worry falling in public), Confined_house (Confined to the house), Washing (Washing), Dressing (Dressing), buttons (Do buttons or shoe laces), Writing (Writing clearly), Cutting (Cutting food), Holddrink (Hold a drink without spilling), Depressed (Depressed), Isolated (Isolated and lonely), Weepy (Weepy or tearful), Angry (Angry or bitter), Anxious (Anxious), Worried (Worried about the future), Felt_conceal (Felt need to conceal PD), Drinking_public (Avoid eating/drinking in public), Embarrassed (Embarrassed due to PD), Worried_reactions (Worried people's reactions), Closerelation (Close relationships), Support_partner (Support from partner), Support_friends (Support from family or friends), fallen_asleep (Unexpectedly fallen asleep), Concentration (Concentration), Poor_memory (Poor memory), hallucinations (Dreams or hallucinations), Speech (Speech), communicate (Unable communicate properly), ignored (Felt ignored), cramps (Painful cramps or spasms), Pain (Pain in joints or body), hot_or_cold (Unpleasantly hot or cold). | | | | | | |

# Supplement Table 5. Confirmatory factor analysis of PDQ-39 items (items arranged according to original description be Peto et al. 1995) with data from COPPADIS study

| **Factor** | **Item** | **Est.** | **Std.-error** | **z-value** | ***P*** | **Lower 96% CI** | **Upper 95% CI** |
| --- | --- | --- | --- | --- | --- | --- | --- |
| Factor 1 | Leisure | 0.777 | 0.021 | 36.642 | < .001 | 0.736 | 0.819 |
|  | Looking | 0.784 | 0.021 | 37.722 | < .001 | 0.743 | 0.824 |
|  | Carry | 0.697 | 0.026 | 26.350 | < .001 | 0.645 | 0.749 |
|  | Walking_mile | 0.857 | 0.017 | 51.727 | < .001 | 0.824 | 0.889 |
|  | Walking_100 | 0.867 | 0.019 | 46.360 | < .001 | 0.830 | 0.904 |
|  | Getting_house | 0.848 | 0.020 | 43.428 | < .001 | 0.809 | 0.886 |
|  | Getting_public | 0.874 | 0.016 | 54.282 | < .001 | 0.842 | 0.905 |
|  | Need_going_out | 0.870 | 0.022 | 38.804 | < .001 | 0.826 | 0.914 |
|  | Worry_falling | 0.760 | 0.026 | 29.578 | < .001 | 0.710 | 0.811 |
|  | Confined_house | 0.802 | 0.024 | 33.850 | < .001 | 0.755 | 0.848 |
| Factor 2 | Washing | 0.907 | 0.018 | 51.174 | < .001 | 0.872 | 0.942 |
|  | Dressing | 0.927 | 0.015 | 59.864 | < .001 | 0.896 | 0.957 |
|  | buttons | 0.752 | 0.023 | 32.059 | < .001 | 0.706 | 0.798 |
|  | Writing | 0.532 | 0.034 | 15.430 | < .001 | 0.465 | 0.600 |
|  | Cutting | 0.795 | 0.024 | 32.708 | < .001 | 0.747 | 0.843 |
|  | Holddrink | 0.719 | 0.037 | 19.378 | < .001 | 0.646 | 0.792 |
| Factor 3 | Depressed | 0.855 | 0.018 | 46.990 | < .001 | 0.820 | 0.891 |
|  | Isolated | 0.892 | 0.023 | 38.183 | < .001 | 0.847 | 0.938 |
|  | Weepy | 0.744 | 0.027 | 27.777 | < .001 | 0.692 | 0.797 |
|  | Angry | 0.798 | 0.024 | 33.773 | < .001 | 0.752 | 0.844 |
|  | Anxious | 0.728 | 0.024 | 30.683 | < .001 | 0.681 | 0.774 |
|  | Worried | 0.577 | 0.033 | 17.257 | < .001 | 0.512 | 0.643 |
| Factor 4 | Felt_conceal | 0.520 | 0.039 | 13.371 | < .001 | 0.443 | 0.596 |
|  | Drinking_public | 0.956 | 0.031 | 30.952 | < .001 | 0.896 | 1.017 |
|  | Embarrassed | 0.854 | 0.026 | 32.783 | < .001 | 0.802 | 0.905 |
|  | Worried_reactions | 0.851 | 0.026 | 32.994 | < .001 | 0.800 | 0.902 |
| Factor 5 | Closerelation | 0.921 | 0.034 | 27.427 | < .001 | 0.855 | 0.987 |
|  | Support_partner | 0.789 | 0.033 | 23.897 | < .001 | 0.724 | 0.854 |
|  | Support_friends | 0.871 | 0.030 | 29.031 | < .001 | 0.812 | 0.930 |
| Factor 6 | fallen_asleep | 0.591 | 0.038 | 15.507 | < .001 | 0.517 | 0.666 |
|  | Concentration | 0.740 | 0.031 | 23.950 | < .001 | 0.679 | 0.801 |
|  | Poor_memory | 0.648 | 0.034 | 19.067 | < .001 | 0.581 | 0.714 |
|  | hallucinations | 0.684 | 0.045 | 15.175 | < .001 | 0.596 | 0.773 |
| Factor 7 | Speech | 0.746 | 0.027 | 27.211 | < .001 | 0.693 | 0.800 |
|  | communicate | 0.834 | 0.028 | 29.993 | < .001 | 0.780 | 0.889 |
|  | ignored | 0.862 | 0.038 | 22.700 | < .001 | 0.788 | 0.937 |
| Factor 8 | cramps | 0.640 | 0.035 | 18.256 | < .001 | 0.571 | 0.709 |
|  | Pain | 0.628 | 0.032 | 19.622 | < .001 | 0.565 | 0.691 |
|  | hot_or_cold | 0.690 | 0.045 | 15.256 | < .001 | 0.601 | 0.778 |
|  | | | | | | | |
| **Chi2 Test** | **Χ²** | **df** | ***p*** |  | | | |
| Baseline Model | 89581.186 | 741 |  |  |  |  |  |
| Factor Model | 1759.658 | 674 | < .001 |  |  |  |  |
| **Fit Indices** | **CFI** | **TLI** | **RMSEA** | **RMSEA 95 % CI lower** | **RMSEA 95 % CI Upper** | **RMSEA *p*** |  |
|  | 0.988 | 0.987 | 0.048 | 0.045 | 0.051 | 0.850 |  |
| Abbreviations: CI = confidence interval, df = Degrees of Freedom, CFI = comparative fit index, TLI = Tucker Lewis Index, RMSEA = root mean squared error of approximation,  Items: Leisure (Leisure activities), Looking (Looking after home), Carry (Carry shopping bags), Walking_mile (Walking half a mile), Walking_100 (Walking 100 yards), Getting_house (Getting around the house), Getting_public (Getting around in public), Need_going_out (Need company when going out), Worry_falling (Worry falling in public), Confined_house (Confined to the house), Washing (Washing), Dressing (Dressing), buttons (Do buttons or shoe laces), Writing (Writing clearly), Cutting (Cutting food), Holddrink (Hold a drink without spilling), Depressed (Depressed), Isolated (Isolated and lonely), Weepy (Weepy or tearful), Angry (Angry or bitter), Anxious (Anxious), Worried (Worried about the future), Felt_conceal (Felt need to conceal PD), Drinking_public (Avoid eating/drinking in public), Embarrassed (Embarrassed due to PD), Worried_reactions (Worried people's reactions), Closerelation (Close relationships), Support_partner (Support from partner), Support_friends (Support from family or friends), fallen_asleep (Unexpectedly fallen asleep), Concentration (Concentration), Poor_memory (Poor memory), hallucinations (Dreams or hallucinations), Speech (Speech), communicate (Unable communicate properly), ignored (Felt ignored), cramps (Painful cramps or spasms), Pain (Pain in joints or body), hot_or_cold (Unpleasantly hot or cold). | | | | | | | |

# Supplement Table 6. Confirmatory factor analysis of PDQ-39 items (items arranged according to original description be Peto et al. 1995, but “ignored” categorized into SOC) with data from COPPADIS study

| **Factor** | **Item** | **Est.** | **Std.-error** | **z-value** | ***P*** | **Lower 96% CI** | **Upper 85% CI** |
| --- | --- | --- | --- | --- | --- | --- | --- |
| Factor 1 | Leisure | 0.777 | 0.021 | 36.651 | <.001 | 0.736 | 0.819 |
|  | Looking | 0.784 | 0.021 | 37.738 | <.001 | 0.743 | 0.825 |
|  | Carry | 0.697 | 0.026 | 26.358 | <.001 | 0.645 | 0.749 |
|  | Walking_mile | 0.857 | 0.017 | 51.715 | <.001 | 0.824 | 0.889 |
|  | Walking_100 | 0.867 | 0.019 | 46.376 | <.001 | 0.830 | 0.903 |
|  | Getting_house | 0.848 | 0.020 | 43.382 | <.001 | 0.809 | 0.886 |
|  | Getting_public | 0.874 | 0.016 | 54.272 | <.001 | 0.842 | 0.905 |
|  | Need_going_out | 0.870 | 0.022 | 38.790 | <.001 | 0.826 | 0.914 |
|  | Worry_falling | 0.760 | 0.026 | 29.583 | <.001 | 0.710 | 0.811 |
|  | Confined_house | 0.802 | 0.024 | 33.847 | <.001 | 0.755 | 0.848 |
| Factor 2 | Washing | 0.907 | 0.018 | 51.202 | <.001 | 0.872 | 0.942 |
|  | Dressing | 0.927 | 0.015 | 59.895 | <.001 | 0.896 | 0.957 |
|  | buttons | 0.752 | 0.023 | 32.111 | <.001 | 0.706 | 0.797 |
|  | Writing | 0.532 | 0.034 | 15.461 | <.001 | 0.465 | 0.600 |
|  | Cutting | 0.795 | 0.024 | 32.696 | <.001 | 0.747 | 0.843 |
|  | Holddrink | 0.718 | 0.037 | 19.356 | <.001 | 0.646 | 0.791 |
| Factor 3 | Depressed | 0.856 | 0.018 | 47.017 | <.001 | 0.820 | 0.891 |
|  | isolated | 0.892 | 0.023 | 38.252 | <.001 | 0.846 | 0.938 |
|  | Weepy | 0.744 | 0.027 | 27.789 | <.001 | 0.692 | 0.797 |
|  | Angry | 0.798 | 0.024 | 33.793 | <.001 | 0.752 | 0.844 |
|  | Anxious | 0.728 | 0.024 | 30.687 | <.001 | 0.681 | 0.774 |
|  | Worried | 0.577 | 0.033 | 17.255 | <.001 | 0.512 | 0.643 |
| Factor 4 | Felt_conceal | 0.520 | 0.039 | 13.378 | <.001 | 0.444 | 0.596 |
|  | Drinking_public | 0.956 | 0.031 | 30.969 | <.001 | 0.896 | 1.017 |
|  | Embarrassed | 0.854 | 0.026 | 32.784 | <.001 | 0.803 | 0.905 |
|  | Worried_reactions | 0.851 | 0.026 | 33.023 | <.001 | 0.800 | 0.901 |
| Factor 5 | Closerelation | 0.886 | 0.032 | 27.778 | <.001 | 0.823 | 0.948 |
|  | Support_partner | 0.768 | 0.034 | 22.564 | <.001 | 0.701 | 0.835 |
|  | Support_friends | 0.845 | 0.030 | 27.713 | <.001 | 0.785 | 0.904 |
|  | ignored | 0.851 | 0.036 | 23.448 | <.001 | 0.780 | 0.923 |
| Factor 6 | fallen_asleep | 0.591 | 0.038 | 15.506 | <.001 | 0.517 | 0.666 |
|  | Concentration | 0.740 | 0.031 | 23.949 | <.001 | 0.679 | 0.800 |
|  | Poor_memory | 0.648 | 0.034 | 19.071 | <.001 | 0.581 | 0.714 |
|  | hallucinations | 0.684 | 0.045 | 15.174 | <.001 | 0.596 | 0.773 |
| Factor 7 | Speech | 0.799 | 0.027 | 30.128 | <.001 | 0.747 | 0.851 |
|  | communicate | 0.913 | 0.029 | 31.165 | <.001 | 0.855 | 0.970 |
| Factor 8 | cramps | 0.640 | 0.035 | 18.243 | <.001 | 0.571 | 0.709 |
|  | Pain | 0.628 | 0.032 | 19.613 | <.001 | 0.565 | 0.691 |
|  | hot_or_cold | 0.690 | 0.045 | 15.266 | <.001 | 0.601 | 0.778 |
|  | | | | | | | |
| **Chi2 Test** | **Χ²** | **df** | ***p*** |  | | | |
| Baseline Model | 89581.186 | 741 |  |  |  |  |  |
| Factor Model | 1690.583 | 674 | <.001 |  |  |  |  |
| **Fit Indices** | **CFI** | **TLI** | **RMSEA** | **RMSEA 90 % CI lower** | **RMSEA 90 % CI Upper** | **RMSEA *p*** |  |
|  | 0.989 | 0.987 | 0.047 | 0.044 | 0.049 | 0.975 |  |
| Abbreviations: CI = confidence interval, df = Degrees of Freedom, CFI = comparative fit index, TLI = Tucker Lewis Index, RMSEA = root mean squared error of approximation,  Items: Leisure (Leisure activities), Looking (Looking after home), Carry (Carry shopping bags), Walking_mile (Walking half a mile), Walking_100 (Walking 100 yards), Getting_house (Getting around the house), Getting_public (Getting around in public), Need_going_out (Need company when going out), Worry_falling (Worry falling in public), Confined_house (Confined to the house), Washing (Washing), Dressing (Dressing), buttons (Do buttons or shoe laces), Writing (Writing clearly), Cutting (Cutting food), Holddrink (Hold a drink without spilling), Depressed (Depressed), Isolated (Isolated and lonely), Weepy (Weepy or tearful), Angry (Angry or bitter), Anxious (Anxious), Worried (Worried about the future), Felt_conceal (Felt need to conceal PD), Drinking_public (Avoid eating/drinking in public), Embarrassed (Embarrassed due to PD), Worried_reactions (Worried people's reactions), Closerelation (Close relationships), Support_partner (Support from partner), Support_friends (Support from family or friends), fallen_asleep (Unexpectedly fallen asleep), Concentration (Concentration), Poor_memory (Poor memory), hallucinations (Dreams or hallucinations), Speech (Speech), communicate (Unable communicate properly), ignored (Felt ignored), cramps (Painful cramps or spasms), Pain (Pain in joints or body), hot_or_cold (Unpleasantly hot or cold). | | | | | | | |

# Supplement Table 7. Confirmatory factor analysis of PDQ-39 items (items arranged according to original description be Peto et al. 1995) with data from Chen at al. 2017

| **Factor** | **Item** | **Est.** | **Std.-error** | **z-value** | ***P*** | **Lower 96% CI** | **Upper 95% CI** |
| --- | --- | --- | --- | --- | --- | --- | --- |
| Factor 1 | Leisure | 0.702 | 0.016 | 42.984 | < .001 | 0.670 | 0.734 |
|  | Looking | 0.754 | 0.016 | 47.166 | < .001 | 0.722 | 0.785 |
|  | Carry | 0.803 | 0.015 | 52.319 | < .001 | 0.773 | 0.833 |
|  | Walking_mile | 0.721 | 0.016 | 44.980 | < .001 | 0.689 | 0.752 |
|  | Walking_100 | 0.818 | 0.017 | 47.601 | < .001 | 0.785 | 0.852 |
|  | Getting_house | 0.864 | 0.015 | 55.904 | < .001 | 0.834 | 0.894 |
|  | Getting_public | 0.877 | 0.014 | 64.133 | < .001 | 0.851 | 0.904 |
|  | Need_going_out | 0.839 | 0.015 | 56.214 | < .001 | 0.810 | 0.868 |
|  | Worry_falling | 0.824 | 0.015 | 54.666 | < .001 | 0.794 | 0.853 |
|  | Confined_house | 0.873 | 0.016 | 54.152 | < .001 | 0.841 | 0.905 |
| Factor 2 | Washing | 0.944 | 0.018 | 51.986 | < .001 | 0.909 | 0.980 |
|  | Dressing | 0.900 | 0.016 | 57.034 | < .001 | 0.869 | 0.931 |
|  | buttons | 0.888 | 0.016 | 56.054 | < .001 | 0.857 | 0.919 |
|  | Writing | 0.623 | 0.018 | 34.273 | < .001 | 0.588 | 0.659 |
|  | Cutting | 0.703 | 0.018 | 38.793 | < .001 | 0.668 | 0.739 |
|  | Holddrink | 0.791 | 0.020 | 40.401 | < .001 | 0.752 | 0.829 |
| Factor 3 | Depressed | 0.906 | 0.013 | 72.179 | < .001 | 0.881 | 0.930 |
|  | Isolated | 0.903 | 0.013 | 67.860 | < .001 | 0.877 | 0.929 |
|  | Weepy | 0.714 | 0.016 | 44.322 | < .001 | 0.682 | 0.745 |
|  | Angry | 0.866 | 0.014 | 60.426 | < .001 | 0.837 | 0.894 |
|  | Anxious | 0.832 | 0.014 | 59.326 | < .001 | 0.804 | 0.859 |
|  | Worried | 0.797 | 0.015 | 54.206 | < .001 | 0.768 | 0.826 |
| Factor 4 | Felt_conceal | 0.579 | 0.020 | 29.046 | < .001 | 0.540 | 0.618 |
|  | Drinking_public | 0.840 | 0.021 | 40.486 | < .001 | 0.800 | 0.881 |
|  | Embarrassed | 0.914 | 0.018 | 51.373 | < .001 | 0.879 | 0.949 |
|  | Worried_reactions | 0.876 | 0.018 | 48.679 | < .001 | 0.841 | 0.912 |
| Factor 5 | Closerelation | 0.874 | 0.031 | 28.552 | < .001 | 0.814 | 0.934 |
|  | Support_partner | 0.614 | 0.027 | 22.543 | < .001 | 0.560 | 0.667 |
|  | Support_friends | 0.867 | 0.030 | 28.686 | < .001 | 0.808 | 0.926 |
| Factor 6 | fallen_asleep | 0.608 | 0.026 | 23.388 | < .001 | 0.557 | 0.659 |
|  | Concentration | 0.688 | 0.025 | 27.127 | < .001 | 0.638 | 0.737 |
|  | Poor_memory | 0.661 | 0.024 | 27.053 | < .001 | 0.613 | 0.709 |
|  | hallucinations | 0.624 | 0.025 | 24.660 | < .001 | 0.574 | 0.674 |
| Factor 7 | Speech | 0.780 | 0.020 | 39.750 | < .001 | 0.742 | 0.819 |
|  | communicate | 0.904 | 0.020 | 44.912 | < .001 | 0.865 | 0.944 |
|  | ignored | 0.807 | 0.021 | 38.745 | < .001 | 0.766 | 0.848 |
| Factor 8 | cramps | 0.772 | 0.023 | 33.613 | < .001 | 0.727 | 0.817 |
|  | Pain | 0.699 | 0.022 | 32.211 | < .001 | 0.656 | 0.742 |
|  | hot_or_cold | 0.865 | 0.027 | 32.258 | < .001 | 0.812 | 0.918 |
|  | | | | | | | |
| **Chi2 Test** | **Χ²** | **df** | ***p*** |  | | | |
| Baseline Model | 50840.219 | 741 |  |  |  |  |  |
| Factor Model | 1201.861 | 674 | < .001 |  |  |  |  |
| **Fit Indices** | **CFI** | **TLI** | **RMSEA** | **RMSEA 95 % CI lower** | **RMSEA 95 % CI Upper** | **RMSEA *p*** |  |
|  | 0.989 | 0.988 | 0.053 | 0.048 | 0.058 | 0.162 |  |
| Abbreviations: CI = confidence interval, df = Degrees of Freedom, CFI = comparative fit index, TLI = Tucker Lewis Index, RMSEA = root mean squared error of approximation,  Items: Leisure (Leisure activities), Looking (Looking after home), Carry (Carry shopping bags), Walking_mile (Walking half a mile), Walking_100 (Walking 100 yards), Getting_house (Getting around the house), Getting_public (Getting around in public), Need_going_out (Need company when going out), Worry_falling (Worry falling in public), Confined_house (Confined to the house), Washing (Washing), Dressing (Dressing), buttons (Do buttons or shoe laces), Writing (Writing clearly), Cutting (Cutting food), Holddrink (Hold a drink without spilling), Depressed (Depressed), Isolated (Isolated and lonely), Weepy (Weepy or tearful), Angry (Angry or bitter), Anxious (Anxious), Worried (Worried about the future), Felt_conceal (Felt need to conceal PD), Drinking_public (Avoid eating/drinking in public), Embarrassed (Embarrassed due to PD), Worried_reactions (Worried people's reactions), Closerelation (Close relationships), Support_partner (Support from partner), Support_friends (Support from family or friends), fallen_asleep (Unexpectedly fallen asleep), Concentration (Concentration), Poor_memory (Poor memory), hallucinations (Dreams or hallucinations), Speech (Speech), communicate (Unable communicate properly), ignored (Felt ignored), cramps (Painful cramps or spasms), Pain (Pain in joints or body), hot_or_cold (Unpleasantly hot or cold). | | | | | | | |

# Supplement Table 8. Confirmatory factor analysis of PDQ-39 items (items arranged according to original description be Peto et al. 1995, but “ignored” categorized into SOC) with data from Chen et al. 2017

| **Factor** | **Item** | **Est.** | **Std.-error** | **z-value** | ***P*** | **Lower 96% CI** | **Upper 95% CI** |
| --- | --- | --- | --- | --- | --- | --- | --- |
| Factor 1 | Leisure | 0.702 | 0.016 | 42.994 | < .001 | 0.670 | 0.734 |
|  | Looking | 0.753 | 0.016 | 47.176 | < .001 | 0.722 | 0.785 |
|  | Carry | 0.803 | 0.015 | 52.323 | < .001 | 0.773 | 0.833 |
|  | Walking_mile | 0.721 | 0.016 | 44.985 | < .001 | 0.689 | 0.752 |
|  | Walking_100 | 0.818 | 0.017 | 47.601 | < .001 | 0.784 | 0.852 |
|  | Getting_house | 0.864 | 0.015 | 55.899 | < .001 | 0.834 | 0.894 |
|  | Getting_public | 0.878 | 0.014 | 64.130 | < .001 | 0.851 | 0.904 |
|  | Need_going_out | 0.839 | 0.015 | 56.221 | < .001 | 0.810 | 0.868 |
|  | Worry_falling | 0.824 | 0.015 | 54.681 | < .001 | 0.794 | 0.853 |
|  | Confined_house | 0.873 | 0.016 | 54.155 | < .001 | 0.842 | 0.905 |
| Factor 2 | Washing | 0.944 | 0.018 | 51.975 | < .001 | 0.908 | 0.980 |
|  | Dressing | 0.900 | 0.016 | 57.060 | < .001 | 0.870 | 0.931 |
|  | buttons | 0.889 | 0.016 | 56.069 | < .001 | 0.585 | 0.920 |
|  | Writing | 0.624 | 0.018 | 34.320 | < .001 | 0.588 | 0.659 |
|  | Cutting | 0.703 | 0.018 | 38.827 | < .001 | 0.668 | 0.739 |
|  | Holddrink | 0.790 | 0.020 | 40.417 | < .001 | 0.752 | 0.828 |
| Factor 3 | Depressed | 0.906 | 0.013 | 72.156 | < .001 | 0.881 | 0.931 |
|  | Isolated | 0.902 | 0.013 | 67.869 | < .001 | 0.876 | 0.928 |
|  | Weepy | 0.713 | 0.016 | 44.327 | < .001 | 0.682 | 0.745 |
|  | Angry | 0.865 | 0.014 | 60.437 | < .001 | 0.837 | 0.893 |
|  | Anxious | 0.832 | 0.014 | 59.326 | < .001 | 0.804 | 0.859 |
|  | Worried | 0.798 | 0.015 | 54.229 | < .001 | 0.769 | 0.826 |
| Factor 4 | Felt_conceal | 0.579 | 0.020 | 29.059 | < .001 | 0.540 | 0.618 |
|  | Drinking_public | 0.840 | 0.021 | 40.472 | < .001 | 0.799 | 0.881 |
|  | Embarrassed | 0.914 | 0.018 | 51.376 | < .001 | 0.879 | 0.949 |
|  | Worried_reactions | 0.876 | 0.018 | 48.680 | < .001 | 0.841 | 0.912 |
| Factor 5 | Closerelation | 0.805 | 0.023 | 35.696 | < .001 | 0.760 | 0.849 |
|  | Support_partner | 0.574 | 0.024 | 23.858 | < .001 | 0.527 | 0.621 |
|  | Support_friends | 0.798 | 0.023 | 35.323 | < .001 | 0.754 | 0.842 |
|  | ignored | 0.892 | 0.022 | 40.277 | <.001 | 0.848 | 0.935 |
| Factor 6 | fallen_asleep | 0.609 | 0.026 | 23.400 | < .001 | 0.558 | 0.660 |
|  | Concentration | 0.688 | 0.025 | 27.115 | < .001 | 0.638 | 0.737 |
|  | Poor_memory | 0.660 | 0.024 | 27.038 | < .001 | 0.612 | 0.708 |
|  | hallucinations | 0.624 | 0.025 | 24.649 | < .001 | 0.574 | 0.673 |
| Factor 7 | Speech | 0.712 | 0.022 | 37.081 | < .001 | 0.769 | 0.845 |
|  | communicate | 0.959 | 0.026 | 37.081 | < .001 | 0.906 | 1.007 |
| Factor 8 | cramps | 0.772 | 0.023 | 33.612 | < .001 | 0.727 | 0.817 |
|  | Pain | 0.699 | 0.022 | 32.216 | < .001 | 0.657 | 0.742 |
|  | hot_or_cold | 0.865 | 0.027 | 32.249 | < .001 | 0.812 | 0.917 |
|  | | | | | | | |
| **Chi2 Test** | **Χ²** | **df** | ***p*** |  | | | |
| Baseline Model | 50840.219 | 741 |  |  |  |  |  |
| Factor Model | 1155.047 | 674 | < .001 |  |  |  |  |
| **Fit Indices** | **CFI** | **TLI** | **RMSEA** | **RMSEA 95 % CI lower** | **RMSEA 95 % CI Upper** | **RMSEA *p*** |  |
|  | 0.990 | 0.989 | 0.050 | 0.046 | 0.055 | 0.430 |  |
| Abbreviations: CI = confidence interval, df = Degrees of Freedom, CFI = comparative fit index, TLI = Tucker Lewis Index, RMSEA = root mean squared error of approximation,  Items: Leisure (Leisure activities), Looking (Looking after home), Carry (Carry shopping bags), Walking_mile (Walking half a mile), Walking_100 (Walking 100 yards), Getting_house (Getting around the house), Getting_public (Getting around in public), Need_going_out (Need company when going out), Worry_falling (Worry falling in public), Confined_house (Confined to the house), Washing (Washing), Dressing (Dressing), buttons (Do buttons or shoe laces), Writing (Writing clearly), Cutting (Cutting food), Holddrink (Hold a drink without spilling), Depressed (Depressed), Isolated (Isolated and lonely), Weepy (Weepy or tearful), Angry (Angry or bitter), Anxious (Anxious), Worried (Worried about the future), Felt_conceal (Felt need to conceal PD), Drinking_public (Avoid eating/drinking in public), Embarrassed (Embarrassed due to PD), Worried_reactions (Worried people's reactions), Closerelation (Close relationships), Support_partner (Support from partner), Support_friends (Support from family or friends), fallen_asleep (Unexpectedly fallen asleep), Concentration (Concentration), Poor_memory (Poor memory), hallucinations (Dreams or hallucinations), Speech (Speech), communicate (Unable communicate properly), ignored (Felt ignored), cramps (Painful cramps or spasms), Pain (Pain in joints or body), hot_or_cold (Unpleasantly hot or cold). | | | | | | | |

# References

1. Epskamp, S., Borsboom, D. & Fried, E. I. Estimating psychological networks and their accuracy: A tutorial paper. *Behav Res Methods* **50**, 195–212 (2018).

2. Peto, V., Jenkinson, C., Fitzpatrick, R. & Greenhall, R. The development and validation of a short measure of functioning and well being for individuals with Parkinson’s disease. *Qual Life Res* **4**, 241–248 (1995).
